# Mitochondrial DNAs provide insight into trypanosome phylogeny and molecular evolution

**DOI:** 10.1186/s12862-020-01701-9

**Published:** 2020-12-09

**Authors:** C. Kay, T. A. Williams, W. Gibson

**Affiliations:** School of Biological Sciences, University of Bristol, Bristol, UK

**Keywords:** Trypanosome, Kinetoplast, Maxicircle, Mitochondrial DNA, Phylogeny, RNA editing

## Abstract

**Background:**

Trypanosomes are single-celled eukaryotic parasites characterised by the unique biology of their mitochondrial DNA. African livestock trypanosomes impose a major burden on agriculture across sub-Saharan Africa, but are poorly understood compared to those that cause sleeping sickness and Chagas disease in humans. Here we explore the potential of the maxicircle, a component of trypanosome mitochondrial DNA to study the evolutionary history of trypanosomes.

**Results:**

We used long-read sequencing to completely assemble maxicircle mitochondrial DNA from four previously uncharacterized African trypanosomes, and leveraged these assemblies to scaffold and assemble a further 103 trypanosome maxicircle gene coding regions from published short-read data. While synteny was largely conserved, there were repeated, independent losses of Complex I genes. Comparison of pre-edited and non-edited genes revealed the impact of RNA editing on nucleotide composition, with non-edited genes approaching the limits of GC loss. African tsetse-transmitted trypanosomes showed high levels of RNA editing compared to other trypanosomes. The gene coding regions of maxicircle mitochondrial DNAs were used to construct time-resolved phylogenetic trees, revealing deep divergence events among isolates of the pathogens *Trypanosoma brucei* and *T. congolense*.

**Conclusions:**

Our data represents a new resource for experimental and evolutionary analyses of trypanosome phylogeny, molecular evolution and function. Molecular clock analyses yielded a timescale for trypanosome evolution congruent with major biogeographical events in Africa and revealed the recent emergence of *Trypanosoma brucei gambiense* and *T. equiperdum*, major human and animal pathogens.

## Background

Trypanosomes are a group of single-celled eukaryotic flagellates, including important pathogens of humans and their livestock (*Trypanosoma* and *Leishmania*), plants (*Phytomonas*) and insects (*Crithidia*). A distinctive feature of trypanosomes is the compartmentalization of the mitochondrial DNA into an organelle located at the proximal end of the flagellum, the kinetoplast, which contains a network of interlocked circular DNAs of two types: maxicircles which are equivalent to the mitochondrial genome of other eukaryotes, and minicircles that encode guide RNAs (gRNAs) used to edit the maxicircle transcripts [1, 2]. Thus both mini- and maxicircles are essential for expression of mitochondrial genes. In trypanosomes, mitochondrial transcripts are edited by the insertion or deletion of uridine residues at positions demarcated by gRNAs to yield mRNAs that can be correctly translated [3–5]. Why this energetically costly and potentially error prone mRNA processing step evolved, and how, are unanswered questions in trypanosome biology, but RNA editing is found throughout the Kinetoplastea [1, 6].

Mitochondrial DNA is widely used in evolutionary, phylogenetic and population genetics analyses and has proved particularly useful as a molecular clock to date speciation events, but the extensive RNA editing of the trypanosome maxicircle might potentially undermine it’s use. Within the Kinetoplastea, trypanosomes are monophyletic according to phylogenetic trees constructed from nuclear-encoded 18S ribosomal RNA (rRNA) and glycosomal GAPDH genes [7, 8], but it has proved difficult to date the emergence of particular lineages, as trypanosomes have no fossil record and are not sufficiently host specific to allow dating by co-speciation with their hosts. Nevertheless, the divergence date of two major groups of pathogenic trypanosomes in Africa (*T. brucei* clade) and South America (*T. cruzi* clade) has been linked to the breakup of Gondwana during the Cretaceous, ~ 100 Mya [7]. The *T. brucei* clade comprises the Salivaria, trypanosomes transmitted via the mouthparts of bloodsucking tsetse flies (*Glossina*) in sub-Saharan Africa, while the *T. cruzi* clade contains the agent of Chagas disease, *T. cruzi*, and related New World trypanosomes [9]. The 100 Mya date has been used to calibrate subsequent trees, e.g. Lewis et al. [10] estimated that *T. cruzi* lineages radiated 3.35 Mya and dated the emergence of two hybrid lineages of *T. cruzi* to < 60,000 years ago. However, the discovery of trypanosomes from wild animals in Africa that belong to the *T. cruzi* clade suggested the possibility of intercontinental transfer more recently via bats or rodents [11] so dating the emergence of trypanosome lineages remains uncertain. A means to infer origins independent of sparse historical information would give valuable insights into the emergence of different pathogens, as well as provide information on how quickly trypanosomes can switch hosts and vectors, with implications for the emergence of new diseases.

Here we have examined the potential of the trypanosome maxicircle for phylogenetic inference and dating. We used long-read sequencing to assemble complete maxicircles from four previously uncharacterized African trypanosomes, including the repetitive non-coding variable region. These assemblies were leveraged to scaffold and assemble a further 103 maxicircle gene coding regions, exploiting the wealth of published short read data. We show that time-resolved phylogenetic trees based on the maxicircle genecoding region can be used to explore events in the recent history of *Trypanosoma brucei* and *T. congolense*, and infer ages which fit well with historical evidence. Our analyses of the pre-edited and non-edited maxicircle genes indicate very high levels of RNA editing in salivarian trypanosomes, limiting further evolution in this direction without incurring functional costs.

## Results

### Whole maxicircle sequences

We sequenced (PacBio Sequel) mitochondrial DNA from four African trypanosomes (*T. congolense* savannah and kilifi, *T. simiae*, and *T. godfreyi*), and assembled complete maxicircles (including the variable region, Additional file 1: Table S2) using the long-read assemblers Canu and Flye [12, 13]. These novel data include the first complete maxicircles for *T. simiae*, *T. godfreyi* and the divergent *T. congolense* kilifi subgroup. We assembled two additional complete maxicircles for the genome strains *T. congolense* IL3000 and *T. vivax* Y486 from published data. We then assembled a further 101 maxicircle gene coding regions from public genome sequence data, using reference sequences or our new assemblies to recover maxicircle reads. In total, we obtained 51 complete maxicircle coding regions for *Trypanosoma brucei,* 34 for *T. congolense,* 3 for *T. equiperdum,* 2 for *T. godfreyi,* 1 for *T. grayi,* 2 for *T. simiae,* and 14 for *T. vivax* (Additional file 2: Table S1)*.* No significant heteroplasmy was detected during sequence assembly.

Complete maxicircles ranged between 19.8 kbp (*T. vivax* Y486) and 27.6 kbp (*T. congolense* IL3000), with most of the size variation occurring in the variable region (4.6 kbp in *T. vivax* Y486 to 12.6 kbp in *T. congolense* IL3000; Additional file 1: Table S2). The overall GC content was 20.9–23.7%, but the GC% of the variable region was much lower (14.1% *T. godfreyi* KEN7 to 17.2% in *T. vivax* Y486). No significant correlation was found between gene coding and variable region GC% (n = 6, ρ = − 0.20, *P* = 0.70), suggesting that changes to the composition of the variable/gene coding regions are independent. Dot plots of the variable regions typically showed two domains: one densely repetitive with short repeats and the other with longer period self-similarity (Additional file 3: Figure S2). Whilst the organisation of the variable region was similar between isolates of the same species (*T. vivax*, *T. congolense*), variation was seen in the fine structure and repeat copy number.

Complete maxicircles were identified by the assembly of circular sequences. For PacBio data, individual reads spanning the entire maxicircle (Additional file 4: Figure S1) were used to validate the assembled sequence.

Reference sequences for the complete variable region of *T. vivax* MT1 as well as the truncated (red line) variable region for *T. b. brucei* Lister 427, are shown to scale against other assembled salivarian variable regions.

### Independent deletions of Complex I genes

**Fig. 1:**
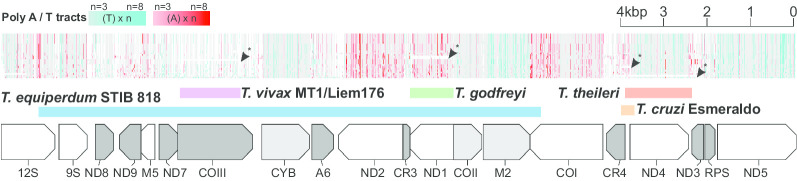
Global overview of maxicircle gene coding region. Top: alignment of the maxicircle mitochondrial DNA gene coding regions from 27 isolates (top to bottom, *Trypanosoma brucei* H866, 1829 ALJO, Lister 427, TREU 927, TSW 55, J10, LF1; *T. congolense* IL3000, WG81, GAM2, IL3900, IL3578, ERA D1; *T. simiae* ERA C2; *T. godfreyi* KEN7, ERA F1; *T. vivax* Liem 176, Y486, Tv2323, *T. cruzi*, CL Brener, Esmeraldo; *T. lewisi*, *T. conorhini*, T*. copemani*, *T. grayi*, *T. theileri*, *Leishmania tarentolae*). Tracts of poly-T or poly-A are shown coloured turquoise or red respectively. An approximate scale is shown. Segmental gene deletions in the alignment are indicated by arrows and are also shown below as coloured bars; the deletion from *T. equiperdum* STIB 818 is also shown for comparison. Bottom: gene order in the maxicircle gene coding region. Non-edited genes in are shown in white, minimally edited genes in light grey and extensively edited (pan-edited) genes in grey. Editing categories are on the basis of *T. vivax* [14]

Alignment of the gene coding regions showed overall conservation of synteny (Fig. 1); however, major gene deletions were evident in *T. godfreyi* (ND1) and *T. theileri* (ND4), as well as previously described deletions in New World *T. vivax *[14]*, T. cruzi* [15]*,* and *T. equiperdum* [16]. The deletion of ND1 in *T. godfreyi* was surprising, as this species undergoes full cyclical development in tsetse flies unlike New World *T. vivax* and *T. equiperdum*, which have both adapted to non-tsetse transmission and evidently do not require a fully functional mitochondrion. The deletion of ND4 in *T. theileri* has also eroded neighbouring genes, CR4 and ND3. Like *T. godfreyi*, *T. theileri* is predicted to require a functional mitochondrion as it completes development to mammal-infective forms in the gut of tabanid flies [17]. One possibility is that these deletions represent an early stage of mtDNA reduction in which mitochondrial function is reduced but not abolished.

Discounting sequences with segmental gene deletions, the size of the region containing both pre-edited and non edited genes (whole coding region, WCR) showed variation across *Trypanosoma* (Table 1) and trypanosome WCRs were approximately 1 kbp smaller than the reference sequences for related trypanosomatids *Crithidia* and *Leishmania*. Among the salivarian trypanosomes (lower portion of Table 1), gene coding regions without deletions (n = 10) are significantly smaller (Kruskal–Wallis one-way ANOVA on ranks, H = 8.0, *P* = 0.05) than those of non-salivarian trypanosomes (n = 4). These size differences can be traced to changes in the length of the pre-edited genes, for if they are summed (Table 1 ‘Σedited’) and subtracted from the whole (Table 1** ‘**WCR-Σedited’), the remaining region is relatively invariant in length (~ 12.4 kbp).

**Table 1:**
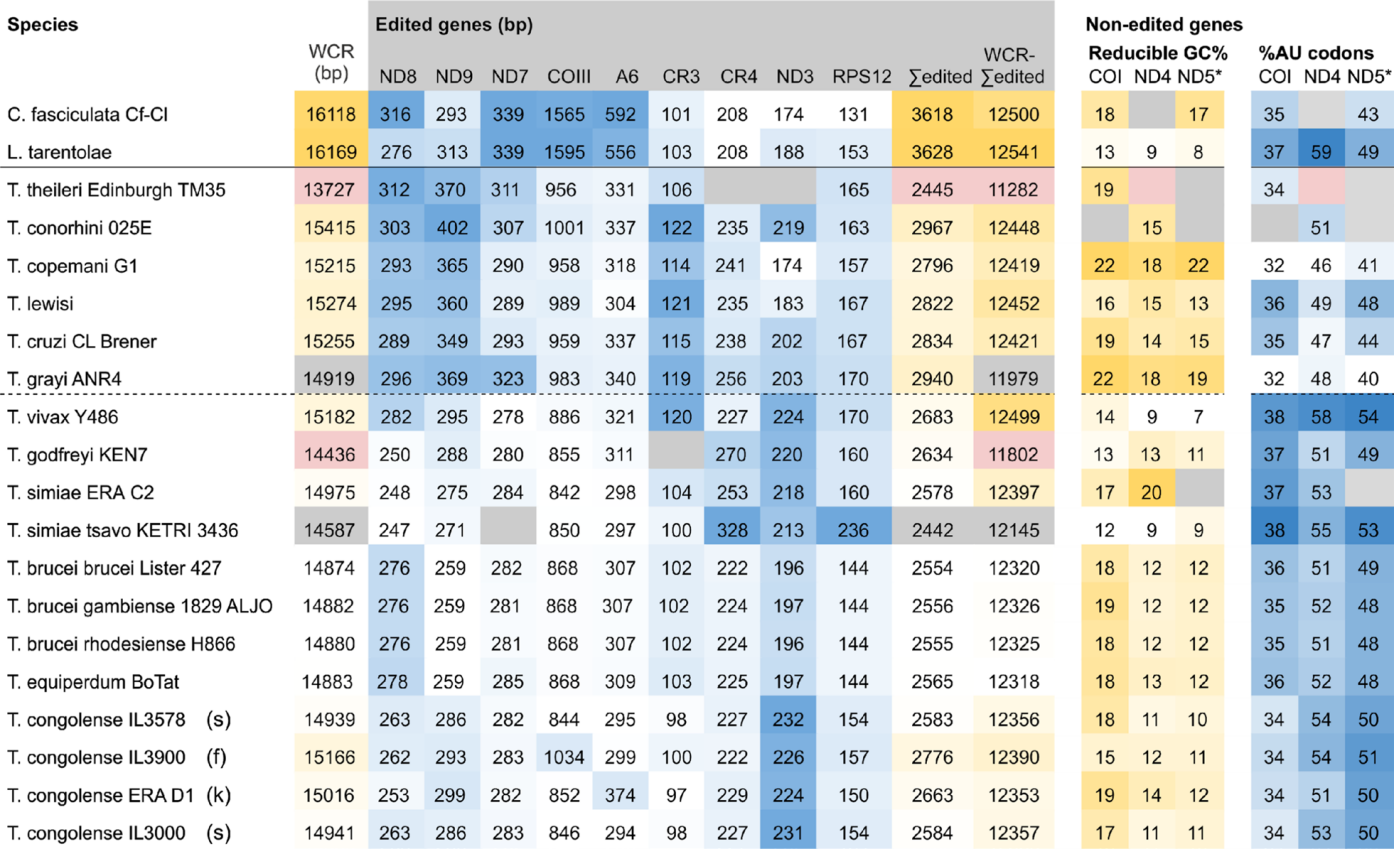
Characteristics of pre-edited and non-edited mitochondrial maxicircle genes in trypanosomatids

Left: Variation in coding region size relates to the sequence contribution of pre-edited genes. Trypanosome gene coding regions (WCR) are shorter than for related trypanosomatids Leishmania and Crithidia. These size differences reflect the contraction of pre-edited regions (Σedited); the remaining gene coding region (WCR-Σedited) is relatively invariant in length. All numbers present the base length of ungapped sequences. Right: Non-edited genes show trends of GC loss which suggest vulnerability to loss of function. Universally non-edited genes COI, ND4 and the first 500 codons of ND5 (ND5*) were analysed for codon usage (% AU codons) and possible composition changes (reducible GC%), which is the percentage of alternate synonymous codons with reduced GC content. Coding regions which are [  ] or have [  ] that impact calculations are highlighted. Numbers have been shaded by value order on a column by column basis. Trypanosomes below the solid line, with non-salivarian above and salivarian below the dashed line.

Gene coding regions frequently contained long homopolymers of either A or T, which appear to relate to reading direction of the gene (Fig. 1), indicating a strand specific bias. In contrast, the untranslated rRNA genes have low GC content but no directional bias in poly-A/T. Comparatively little expansion and contraction was observed in the intergenic regions, although in *T. vivax* a putative microsatellite was identified between the 9S and ND8 genes (Additional file 5: Figure S3).

### High levels of RNA editing in salivarian trypanosomes

The overall GC% of the maxicircle gene coding region was low (~ 25%) in trypanosomes and other trypanosomatids, but these mean values conceal the fact that pre-edited genes are far more GC-rich than non-edited genes (Table 2). Comparison of these genes indicates that they are significantly more GC-rich in salivarian compared to non-salivarian trypanosomes (**ND8**, **ND9**, **COIII**, **A6** (n = 12,6); **CR3**, **ND7** (n = 11,6); Kruskal–Wallis one-way ANOVA on ranks all H > 14, P = < 0.001). These genes also show variation in T:C ratios, with particularly high T:C ratios in ND9, CR3 and CR4 (Table 2).

**Table 2:**
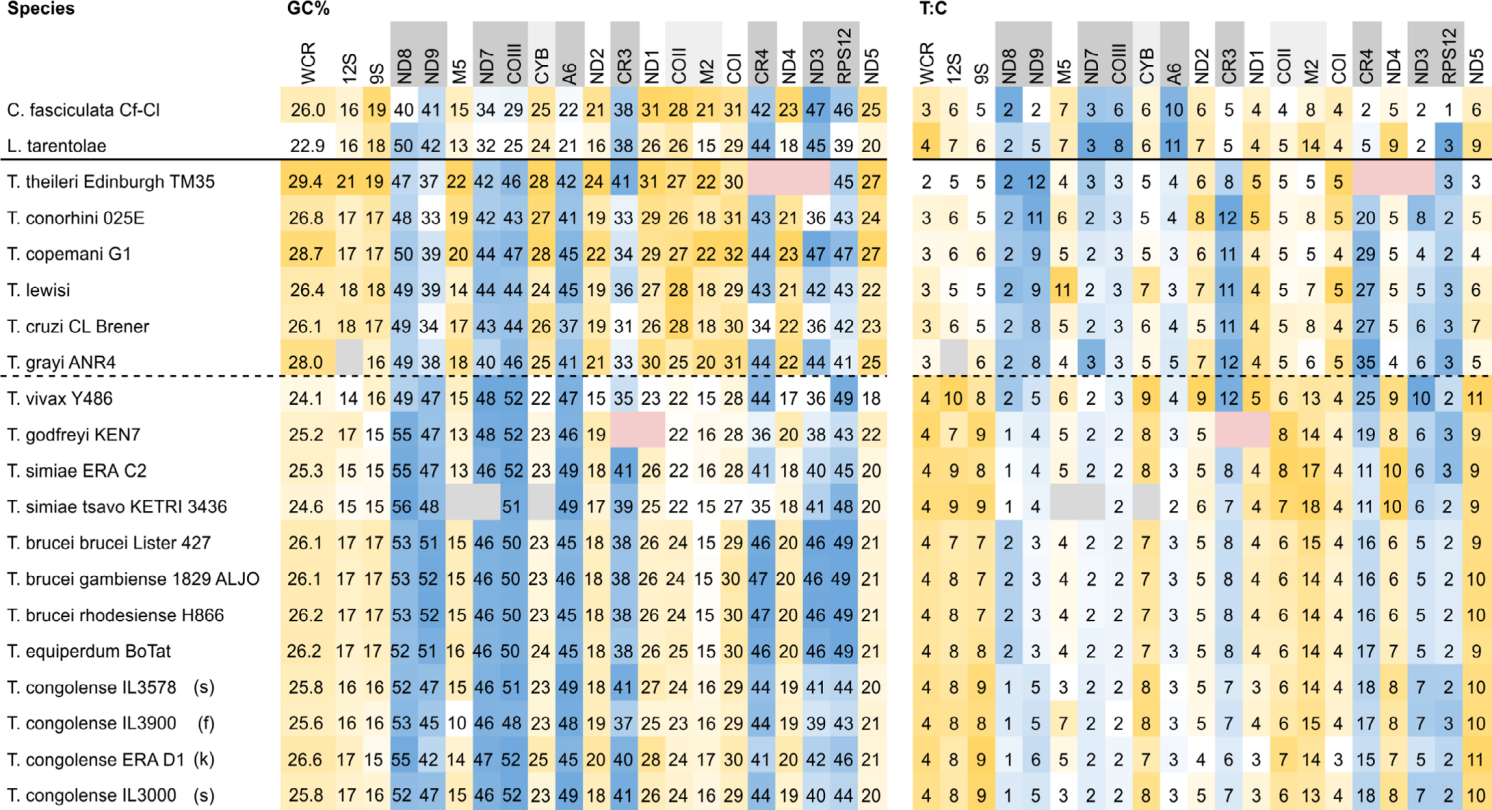
Individual maxicircle genes show different trends for GC composition and T:C ratios

From an alignment of gene coding regions, aligned sequence regions were extracted and analysed in the reading direction for GC% (left) and the ratio of T:C (right). Extensively edited genes ([  ]) have greater GC% than lightly ([  ]) or non-edited (white) genes. Likewise, the T:C ratio is very high in some edited genes e.g. ND9, CR3, CR4. Shading as Table 1.

**Fig. 2:**
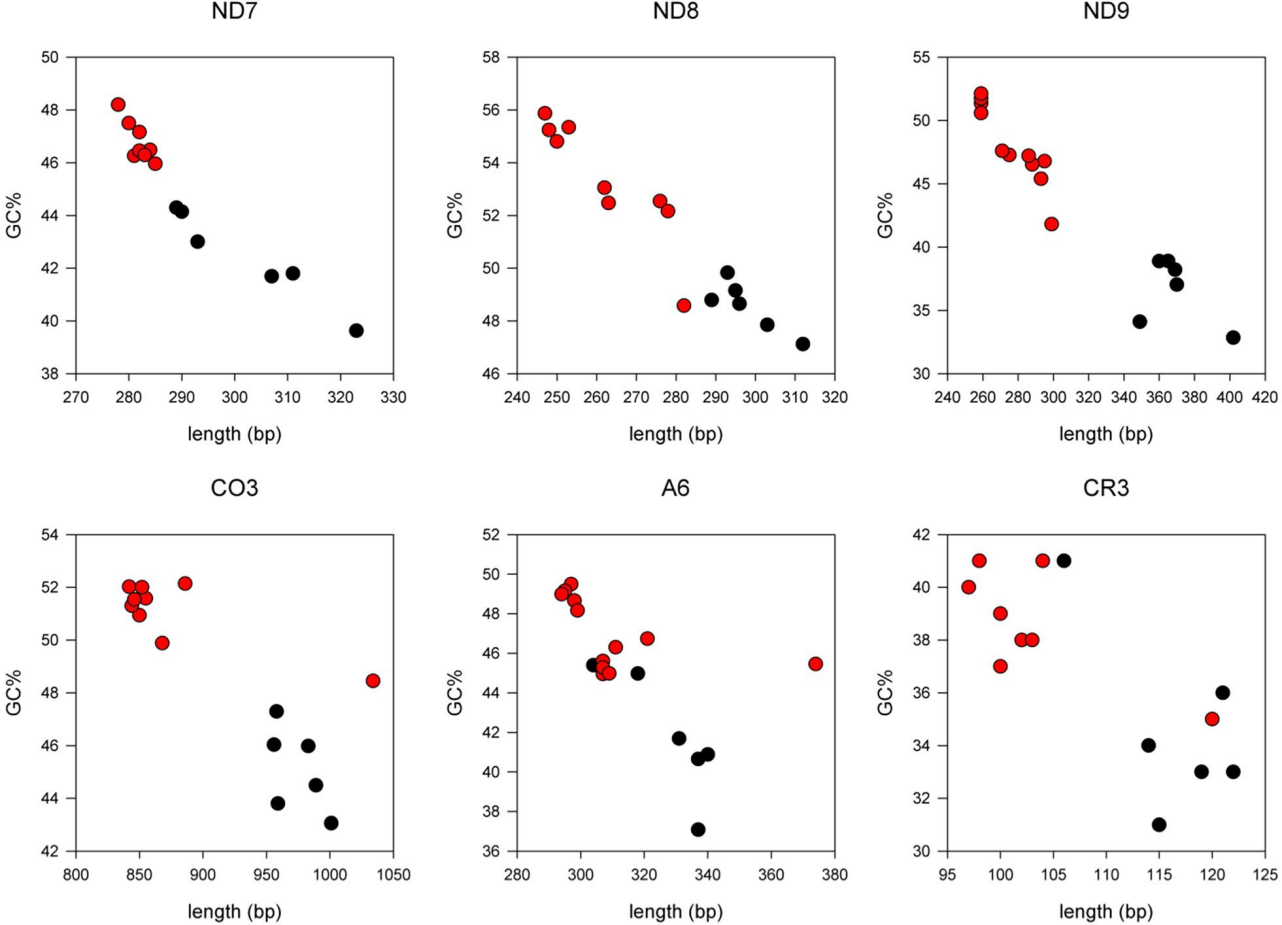
Correlation between sequence length and GC% for six pre-edited maxicircle genes. There is an inverse correlation between the length and GC% of pre-edited genes in trypanosome maxicircles, where salivarian trypanosomes (red filled circle) generally have shorter, more GC-rich pre-edited genes than non-salivarian trypanosomes (black filled circle).This relationship also holds true for other trypanosomatid species (Additional file 6: Figure S4)

Some pre-edited genes showed a large variation of sequence length (Table 2), which was inversely proportional to GC% (ρ = **ND8**_(n=20)_ − 0.91, **ND9**_(n=20)_ − 0.93, **ND7**_(n=19)_ − 0.98, **COIII**_(n=20)_ − 0.96, **A6**_(n=20)_ − 0.95, **CR3**_(n=19)_ − 0.80, all *P* = < 0.001, Fig. 2, Additional file 6: Figure S4). Analysis of base composition reveals a strong proportional correlation of sequence length to T% (Additional file 6: Figure S4) whilst A% has a weaker inverse correlation (ρ = A%/T%, **ND8**_(n=20)_ − 0.72/0.98, **ND9**_(n=20)_ − 0.65/0.96, **ND7**_(n=19)_ − 0.72/0.99, **COIII**_(n=20)_ − 0.84/0.97, **A6**_(n=20)_ − 0.79/0.96, **CR3**_(n=19)_ − 0.75/0.97, all *P* = < 0.005). Providing that the translated product remains similar in size, shorter genes indicate a greater extent of editing in salivarian compared to non-salivarian trypanosomes; presumably the increase in GC% and decline specifically in T% is offset by U insertion during RNA editing.

### Non-edited genes are approaching limits to GC loss

Unlike pan-edited genes, non-edited and lightly edited genes are characterised by low GC% (Table 2). However the T:C ratios for some genes vary significantly between trypanosomes (Table 2), especially between salivarian and non-salivarian trypanosomes (**12S**_(n=12,5)_, **9S**_(n=12,6)_, **CYB**_(n=11,6)_, **M2**_(n=12,6)_, **COI**_(n=12,6)_ **ND4**_(n=12,5)_, **ND5**_(n=12,6)_, one-way ANOVA all F > 70, *P* < 0.001; **COII**_(n=12,6)_, F = 3.6, *P* = 0.06), whilst other genes do not show significant change (**M5**_(n=11,6)_ F = 0.56, *P* = 0.46; **ND2**_(n=12,6)_ F = 2.92, *P* = 0.10; **ND1**_(n=11,6)_ F = 5.48, *P* = 0.03).

The low GC% of non-edited genes suggests that further reduction might lead to non-synonymous substitutions. Indeed, the total number of AU codons (no G or C) is already high, exceeding 50% in ND4 and ND5, and only a small proportion of remaining codons could be converted to AU codons without incurring translational changes (Table 1). Six amino acids (F, I, K, L, N, Y) solely use AU codons, and for amino acids encoded by GC or AU codons, the AU codon was strongly preferred. Thus further GC loss would either result in non-synonymous mutations or introduce a compositional bias in the gene product, suggesting that non-edited genes have reached the limit of GC loss, particularly in salivarians.

### Time-resolved phylogeny of African trypanosomes

To test whether the discrete mechanisms driving sequence change in the maxicircle gene coding region would affect phylogenetic analysis between species of trypanosomes, alignments were prepared of (a) individual genes, (b) sets of pre-edited and non-edited genes, and (c) the whole gene coding region (WCR) with and without pre-edited genes (Additional file 7: Figure S5). Trees inferred from individual genes (e.g. COI) were congruent, providing no evidence of recombination between maxicircle loci, and strongly supported the monophyly of salivarians, although they showed weak resolution in the topology of deeper branches. Sets of non-edited genes had consistent topology for deeper branches, but were so conserved that the resolving power within species was limited. Topologies inferred from pre-edited regions alone, which as a whole are faster-evolving, resolved intraspecific groups confidently but presented conflicting topologies for deeper branches. Using the entire gene coding redion, including pre-edited genes, gave better resolving power (in terms of bootstrap support) than individual genes or sets of non-edited genes. Therefore, the use of the entire gene coding region including pre-edited genes and intergenic sequence appears to be a useful phylogenetic marker for trypanosome evolution [18] and were used in subsequent analyses.

**Fig. 3:**
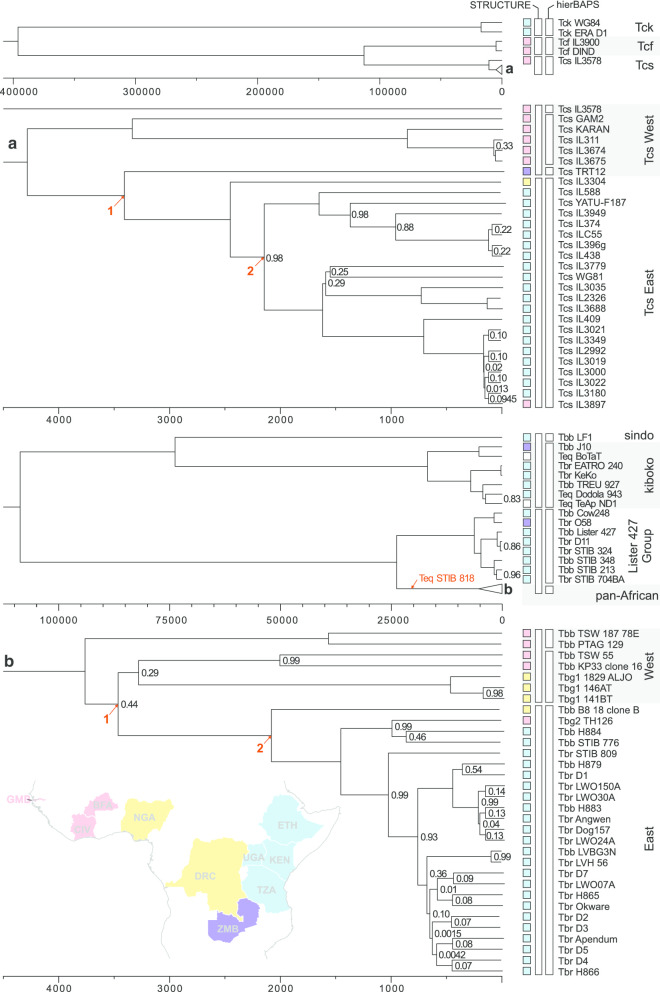
Time resolved phylogenies of *T. congolense* and *T. brucei*. The savannah (**a**) and pan-African (**b**) clades are expanded below their respective trees. The coloured boxes correspond to countries of origin on the map of Africa (inset). STRUCTURE and hierBAPS groups are indicated by the white boxes. Timelines are in years before present and node values are posterior probabilities < 1. Arrowed nodes 1 and 2 are discussed in the text. The putative position of *T. equiperdum* STIB818 inferred from an independent ML tree (Additional file 10: Figure S6) is indicated in **b**

Aligned WCRs of *T. congolense* (including savannah, forest and kilifi subgroups) and *T. brucei* (including *T. equiperdum* strains with complete coding regions) were used to construct time-resolved phylogenies (Fig. 3). To date species trees, the molecular clock was calibrated using tip isolation dates (Additional file 8: Data S1). Best marginal likelihoods were obtained with birth–death models using strict molecular clocks. Clock rates for *T. brucei* (median 1.81 × 10^–7^ substitutions/site/year, s/s/y, 95% HPD interval 1.00 × 10^–6^–5.56 × 10^–7^ s/s/y) and *T. congolense* (median 7.45 × 10^–7^ s/s/y, 95% HPD interval 1.43 × 10^–8^–2.89 × 10^–6^ s/s/y) were found to be similar in magnitude but significantly different from each other (Kruskal–Wallis one-way ANOVA on ranks, H = 680, *P* < 0.001). Clock rates calculated alone for the *T. brucei* pan-African clade and *T. congolense* savannah have similar (median *T. brucei* 2.35 × 10^–7^ s/s/y, *T. congolense* 1.15 × 10^–6^ s/s/y) but significantly faster (Kruskal–Wallis one-way ANOVA on ranks, H > 80, *P* < 0.001) rates compared to the species as a whole (Additional file 9: Data S2). The rates reported here are similar to those reported for other mitochondrial clocks [19], and faster than the estimated rate of trypanosome nuclear evolution based on 18S data (~ 1 × 10^−10^ s/s/y) [20]. Rates calculated from regions with non-edited protein coding genes (ND2 < > COI) have lower substitution rates (median, *T. brucei* 1.62 × 10^−7^ s/s/y, *T. congolense* 4.50 × 10^−7^ s/s/y) compared to the maxicircle gene coding region as a whole. However rates for this region are faster than rates predicted for the *T. cruzi* COII–ND1 region (~ 2 × 10^−8^ s/s/y) [21].

The inferred tree for *T. congolense* shows three clades, deeply separated in time, corresponding to the three known subgroups (Fig. 3). The kilifi subgroup (Tck) diverged approximately 400 kya (95% HPD interval 29–1200 kya), and the forest (Tcf) and savannah (Tcs) subgroups approximately 115 kya (95% HPD interval 20–680 kya). Most isolates fell in the Tcs clade (Fig. 3), which was further subdivided into two clades with a divergence date of ~ 4 kya; these clades broadly comprise East (Kenya, Uganda, Ethiopia and Zambia) and West (The Gambia and Burkina Faso) African isolates. Results from hierBAPS and STRUCTURE analyses confirmed these results, and in addition hierBAPS resolved TRT12 from Zambia and IL3578 from Burkina Faso as separate individuals (Fig. 3); for these analyses, runs with and without admixture models had the same best predicted K, except in the resolution of *T. congolense* subgroups.

The *T. brucei* tree revealed two deeply separated clades, which diverged ~ 108 kya (95% HPD interval 8–325 kya); these two clades also emerged from STRUCTURE analysis (Fig. 3). Separate from the main clade of *T. brucei* subspecies isolates is a clade containing isolates previously identified as belonging to kiboko (J10, 927) and sindo (LF1) groups on the basis of maxicircle restriction fragment length polymorphisms [22] and COI haplotypes [23]; interestingly this clade also contains Old and New World *T. equiperdum* isolates (BoTat, Dodola 943, TeAPND1). The main *T. brucei* sspp. clade is further subdivided, separating a group of East African isolates containing Lister 427 from a pan-African group ~ 23 kya. A group of largely East African isolates emerge from the pan-African clade ~ 3.5 kya, and this group is also present in STRUCTURE and hierBAPS analyses (Fig. 3).

The inferred trees for *T. brucei* give insights into the evolution of *T. equiperdum* and *T. b. gambiense*. For *T. equiperdum* only three isolates with whole coding regions were included in time-resolved analysis, as sequences of other isolates are either incomplete or have large deletions [STIB841 and STIB842 are truncated in ND5; STIB818 has lost most of the maxicircle (Fig. 1)]. The positions of these other isolates were inferred from maximum likelihood trees on shared sequence (partial 12S, partial COI to partial ND5, ~ 4.7 kbp of sequence), which indicate that STIB841 and STIB842 group with the other *T. equiperdum* isolates with full length sequences, while STIB818 is placed separately (Additional file 10: Figure S6). The divergence date for this main group of *T. equiperdum* from *T. b. brucei* t is around 5000 ya (*T. b. brucei* J10 and *T. equiperdum* BoTat 5190 ya (95% HPD interval 360–15,800 ya); *T. b. brucei* 927 and *T. equiperdum* Dodola 943/TeAPND1 4310 ya (95% HPD interval 232–9810 ya)). However, the position of STIB818 suggested by maximum likelihood trees could support a much older origin for this lineage.

Origins of both Type 1 and 2 T*. b. gambiense* (*Tbg1* and *Tbg2*) can be estimated from the inferred trees: *Tbg1* 3,240 ya (95% HPD interval 222–8380 ya); *Tbg2* ~ 1000 ya (95% HPD interval 80–3020 ya). This result is consistent with a published emergence date of 750–9500 years ago for *Tbg1*, based on estimated mutation rates and the observed number of mutations accumulated per genome in this asexual lineage [24].

Despite the difference in clock rates for each species, the time resolved phylogenies indicate that both *T. congolense* and *T. brucei* underwent a major divergence events simultaneously (Fig. 3: 1, West to Central Africa, *T. congolense* 3410 ya, 95% HPD interval 294–13,400 ya; *T. brucei* 3430 ya, 95% HPD interval 236–9610 ya; 2, expansion into East Africa *T. congolense* 2160 ya, 95% HPD interval 160–6800 ya; *T. bruc*ei 2070 ya, 95% HPD interval 141–6210 ya), posing intriguing questions about the causes. Possible reasons include the major climatic changes that have affected the African continent in the past few thousand years, including the gradual desiccation of the Sahara desert (~ 3.0 kya) and the closure of the Dahomey gap (~ 4.5 kya) [25], overlaid by movements of wild animals, humans and their livestock in response to ecological changes.

## Discussion

The trypanosome maxicircle presents a complex evolutionary system, with several discrete mechanisms bringing about sequence change. Synteny in the gene coding region is largely conserved, except for segmental gene deletions in some lineages (*T. equiperdum*, New World *T. vivax*, *T. godfreyi*, *T. theileri*), leading to presumed loss of function. Whether complete maxicircle loss, as seen in *T. evansi*, is the inevitable fate for maxicircles with small deletions remains unclear, but the fact that several maxicircles with deletions have been found suggests that maxicircle loss may not happen as a single event.

Assembled maxicircles have low GC content in both gene coding and variable regions. For non-edited genes the remaining permissive mutational space for further GC loss is small, particularly in salivarians, as few mutations would be synonymous and protein composition might already be compromised. The true extent of GC loss in pre-edited genes is cryptic as additional coding information comes from the minicircle gRNAs, however the declines in pre-edited gene length indicate that genes are more extensively edited in salivarian compared to non-salivarian trypanosomes. Given the recent emergence of the salivarian clade this would conflict with the idea that RNA editing is a primitive kinetoplastid feature that is always “on the way out” [1, 6].

The observed base composition biases in the maxicircle could be driven by the loss of recombination, as GC loss is a feature commonly observed in non-recombining populations [26]. Alternatively base composition biases could reflect the metabolic cost and availability of these nucleobases [27]. In non-edited genes a strand-specific bias for poly-T as well as selection for AU codons suggests that selection acting at the level of the transcript, such as for translational efficiency or against transcript cost, influences the evolution of these sequences. The rRNA genes have low GC content but as they are not translated are not expected to share the same codon selection pressures. The variable region has the lowest GC content, but wide variations in the size even within the same species, suggesting that it is not being streamlined for a reduced cost.

The Salivaria appear as a distinct group in the analyses presented, sharing properties of increased T:C ratio in their non-edited genes and shorter edited genes compared to non-salivarian trypanosomes. This disjunction suggests that the salivarians have undergone a period of evolutionary change, perhaps associated with their adaptation to transmission via the salivary route in tsetse. Unfortunately there are no intermediate taxa to sample. Although *T. grayi* is also transmitted by tsetse, this is by the posterior rather than salivarian route, and *T. grayi* is not a close relative of salivarian trypanosomes in phylogenetic analyses [8, 9, 28].

Despite the different evolutionary processes at work in the maxicircle gene coding region, our analyses demonstrate that it is a useful tool for phylogenetic analysis and a good molecular clock within a species. From population genetics analyses and the consistent phylogeny of isolates using different portions of the maxicircle, recombination appears rare or is restricted to very closely related sequences in the salivarian trypanosomes *T. congolense* and *T. brucei*. This contrasts with *T. cruzi,* where evidence for recombination and heteroplasmy has been presented [29, 30]*.* Our analysis of *T. brucei* and *T. congolense* suggests that the maxicircle can be used to probe the recent history and distribution of a species using isolation dates without other assumptions. The dates inferred for the *T. brucei* group fit well with estimations for the date of emergence of the human pathogen *T. b. gambiense* Type 1, previously calculated as 750–9500 ya, based on estimated mutation rates and the observed number of mutations accumulated per genome in this asexual lineage [24]. These dates fit with the development of settled agriculture and burgeoning centres of population in West Africa in the past 10,000 years that favoured the evolution of parasites adapted to human to human transmission. As shown by previous studies [31], *T. equiperdum* is polyphyletic. A new finding here was the emergence of one clade of *T. equiperdum* from the divergent group of *T. b. brucei* associated with wild animal-tsetse transmission cycles in East Africa, referred to as kiboko/sindo group [22]. This puts a new perspective on the evolution of *T. equiperdum* from *T. b. brucei,* with an estimated emergence date of *T. equiperdum* of ~ 5000 ya. The kiboko/sindo clade itself is estimated to have diverged from the main *T. brucei* clade > 108,000 ya.

Besides the kiboko/sindo clade, a small group of East African *T. b. brucei* and *T. b. rhodesiense* isolates was clearly separate from the majority of *T. brucei* isolates from sub-Saharan Africa. The human pathogen *T. b. rhodesiense* is characterised by a unique gene, the *SRA* (serum resistance associated) gene, which confers the trait of human infectivity [32]. Two major sequence variants of this gene have been identified that distinguish *T. b. rhodesiense* isolates from northern and southern East Africa. Here, *T. b. rhodesiense* LVH 56 (northern *SRA* variant) and *T. b. rhodesiense* 058 (southern *SRA* variant) were found in separate clades in the tree (Fig. 3), with an estimated divergence time of ~ 23,000 ya, placing the emergence of the *SRA* gene, and consequently *T. b. rhodesiense,* earlier than this date.

The dated phylogeny also has ramifications for the evolution of the important livestock pathogen, *T. congolense*. Of the three subgroups of *T. congolense,* kilifi is the earliest diverging, estimated to have split from the forest and savannah subgroups ~ 400,000 ya. *T. congolense* savannah and forest subgroups diverged more recently around 115,000 ya. The more extensive sampling of the savannah subgroup provides evidence of a split between East and West African isolates about 4000 ya. The position of TRT12 from Zambia on a long branch at the edge of the East African clade suggests that further subdivisions may emerge with more sampling of the savannah subgroup throughout its geographical range, as already suggested in other studies [33].

From the difference in calculated clock rates for *T. brucei* and *T. congolense,* it is clear that clock rates vary between trypanosome species, which fits with the observation that the rate of nuclear evolution in salivarians is seven to tenfold higher than non-salivarians [20]. It is also reasonable to assume that there is rate variation across the coding region. At present, the geological timescale of salivarian divergence is poorly constrained, with most published studies based on a single calibration of divergence between New World and Old World trypanosomes at 100 Mya, coincident with the splitting of Africa and South America. However, it is difficult to exclude the possibility that trypanosome exchange between continents might have occurred much more recently [11], which would have a major impact on inferred rates. The alternative of using isolation dates may provide a useful complementary approach for investigating more recent divergences within trypanosome clades. We show here that isolation dates can be used to explore events in the recent history of a species, and infer ages which fit well with historical evidence (*T. equiperdum*, *T. b. gambiense*). Rate calculations for *T. brucei* and *T. congolense* from different sets of isolation dates are in strong agreement for geographically shared events in recent history, and could be tested further by future analysis of *T. vivax*. Future analyses of deeper trypanosome evolution must address assumptions on how rates are calculated, how rate varies between species, and our confidence in using geological events for speciation barriers. This would put us in a better position to understand the evolution of the salivarian trypanosomes and the genus as a whole, and infer accurate dates for the origins of the group.

## Conclusions

The maxicircle data we present represents a new resource for experimental and evolutionary analyses of trypanosome phylogeny, molecular evolution and function. Despite the different evolutionary processes at work in the maxicircle coding region, our analyses demonstrate that it is a useful tool for phylogenetic analysis and a good molecular clock within a species. Molecular clock analyses yielded a timescale for trypanosome evolution congruent with major biogeographical events in Africa and revealed the recent emergence of *Trypanosoma brucei gambiense* and *T. equiperdum*, major human and animal pathogens.

## Methods

### Genomic DNA extraction

High molecular weight DNA for genome sequencing was purified from axenically-grown procyclic trypanosomes using a Blood and cell culture kit (Qiagen) and a modification of the manufacturer’s yeast cell protocol. Briefly, approximately 5 × 10^8^ trypanosomes were pelleted by centrifugation, washed once with PBS and resuspended in 5 ml lysis buffer containing proteinase and RNAase as per the manufacturer’s protocol. Following 1 h incubation at 50 °C, lysates were centrifuged at 5000 rpm for 5 min at room temperature in a microfuge to pellet debris before the supernatant was applied to a Genomic-tip 100/G column (Qiagen). Subsequent processing followed the manufacturer’s protocol; after isopropanol precipitation, DNA was resuspended in 200 µl 10 mM Tris, 0.1 mM EDTA, pH 8 and stored at 4 °C.

### Sequence data

Long read data was obtained on a PacBio Sequel II System, using 1 or 2 cells of a 4 reaction SMRT Cell 1M v2 plate per sample, and prepared using the SMRTbell^®^ Template Prep Kit 1.0. Short read sequence data was obtained on an Illumina NovaSeq producing approximately 20 Gbp of 150 bp paired end reads per sample. Reference sequences were obtained from NCBI Refseq database [34]. Data in the Sequence Read Archive [35] was recovered using the SRA Toolkit [36]. All the data sources used for assembly are listed in Additional file 2: Table S1.

### Assembly

#### Illumina assembly

For species with reference maxicircles, Illumina sequence data was searched using Magic-BLAST v1.4.0 [37] for aligning reads. Pooled reads were then assembled using SPAdes v3.13.1 [38] and maxicircle contigs identified by BLAST v2.2.31+ [39]. Where assembly yielded multiple maxicircle contigs, those of > 1000 bp were oriented and scaffolded using MeDuSa v1.6 [40]. For species without close references (e.g. *T. grayi*) NOVOplasty v3.3 [41] was used to extend the COI seed region of a related species to yield partial maxicircle sequences.

#### PacBio assembly

Long PacBio reads spanning the maxicircle were identified by BLAST; an example maxicircle spanning read is shown in Additional file 4: Figure S1. These reads were then used to fish additional sequences from the read pool. Reads were then corrected using Canu v1.8 [13] and split to less than 12 kbp before being assembled with Flye v2.5 [12]. Illumina read data, where available, were used to polish Flye assembled maxicircles.

#### Sanger assembly

Maxicircle reads were identified by BLAST against a reference maxicircle and assembled using CAP3 [42].

#### Assembly assessment

Reads were aligned to the assembled maxicircle sequences using BWA MEM v0.7.17 [43] and visualised in Tablet v1.19.09.03 [44]. Dot plots were produced in Flexidot v1.06 [45].

#### *T. theileri*

The *T. theileri* maxicircle sequence was identified from the assembled contig pool by BLAST.

Additional information on assembly if given in Additional file 11: Methods S1.

### Gene annotation

For partially assembled maxicircles BLAST was used to recover individual non-edited genes. Complete coding regions were prepared using BLAST to crop assembled maxicircles between 12S rRNA and ND5 genes. Sequences were aligned using MAFFT v7.427 [46] (coding sequence; G-INS-i, PAM 200, k = 2, individual genes); short sequences were discarded. An approximation of gene boundaries for edited genes was made by aligning an annotated coding region of *T. vivax* Y486 to the coding region alignment and cropping sub-alignments on the basis of these annotations. For non-edited protein coding genes, gene boundaries could be determined by predicted open reading frame using translation Table 4 (https://www.ncbi.nlm.nih.gov/Taxonomy/Utils/wprintgc.cgi).

### Phylogenetics

Maximum likelihood trees were inferred using IQ-Tree v1.6.12 [47], using ModelFinder [48] to find the best-fitting nucleotide substitution models. Parameters from these runs were used to inform a time-resolved phylogeny using BEAST2 v2.6.1 [49]. A birth–death model using isolation dates as tip dates and a strict molecular clock was used for both *T. congolense* and *T. brucei*, on the basis of marginal likelihood estimation using the BEAST2 path sampling and ModelTest packages. Each run was sampled every 100 iterations over a chain length of 10,000,000 with the first 10% discarded as burn-in; analyses were examined in Tracer v1.7.1 [50]. Treeannotator v1.10.4 was used to extract a consensus tree from the sampled population, and trees were visualised in FigTree v1.4.2.

### Population genetics

Clustering of isolates into groups was performed by first extracting variable positions from aligned coding regions using SNP-sites [51]. Appropriately formatted files were then prepared using PGDSpider v2.1.1.5 [52] for later use in hierBAPS [53] and STRUCTURE [54]. Job runs in STRUCTURE used 10 iterations of admixture and no admixture models between K 1–8, with 5000 generations of burn-in and 5000 sampled, and assumed the maxicircle as a haploid allele. STRUCTURE HARVESTER v0.6.94 [54, 55] was then used to determine K. Runs in hierBAPS used 4 levels and 20 initial clusters and were run until convergence.

### Statistics

Comparison of sequence properties between species used a representative from each species and species subgroup. For comparing clock rates, 900 evenly sampled clock rates after a 10% burn-in on a MCMC chain length of 10,000,000 were used for the basis of analysis. Where gene or sequence properties are compared, tests for normality (Shapiro–Wilk) and equal variance were first applied to determine an appropriate test of variance (normal, one-way ANOVA; non-normal, Kruskal–Wallis one-way ANOVA on ranks). All correlations used the Pearson correlation coefficient (ρ =).

## Supplementary information

**Additional file 1: Table S2.** Properties of the six complete salivarian maxicircles.

**Additional file 2: Table S1. **Strain information and source data.

**Additional file 3: Figure S2. **Assembled variable regions of salivarian maxicircle mitochondrial DNAs.

**Additional file 4: Figure S1.** An example of a PacBio read spanning the entire sequence of the trypanosome mitochondrial DNA (maxicircle). A single read from the *T. congolense* GAM2 readpool is shown dot-plotted against itself. The highly repetitive short period portion of the variable region is visualised as a densely self-similar region between 20-26 kbp, whilst the longer period portion of the variable region begins at 15 kbp. The remainder of the sequence shown belongs to the gene coding region. The complete length of the maxicircle is seen from 0-26.3 kbp, and thereafter begins to repeat. The assembled sequence is shown in Additional file 3: Figure S2.

**Additional file 5: Figure S3.** Trypanosoma vivax, an alignment of the intergenic region between 9S and ND8 containing a putative microsatellite. Bases are shown as coloured bands with the top line tick showing 20 bp increments. The sequence [ATATA] is tandemly repeated between 18 and 51 times in the selected isolates.

**Additional file 6: Figure S4. **Correlation between sequence length, GC% and T% for six pre-edited maxicircle genes. Some pre-edited maxicircle genes exhibit transcript length variation with strong correlation between length and T% as well as an inverse correlation for GC%. The weak negative correlation for A% indicates that this is a strand specific phenomenon consistent with RNA editing, where uridines are inserted back into the transcript. Key: *Crithidia, Leishmania* (open circle), salivarian (red filled circle) and non-salivarian trypanosomes (black filled circle).

**Additional file 7: Figure S5. **A comparison of maximum likelihood trees inferred from different regions of the trypanosome maxicircle mitochondrial DNA. In general using more sequence contributes to higher bootstrap support for the inferred maximum likelihood topology. If individual genes are used, confidence for the deepest branches is reduced, and topological variances are observed. Collections of non-edited genes have a consistent topology but fail to resolve well within species. Use of the entire gene coding region (WCR), with or without pre-edited genes, provides better supported trees. If pre-edited genes alone are used, structure within species is well supported, but multispecies relationships are poorly resolved.

**Additional file 8: Data S1. **Distribution of isolation dates used for inferring time-resolved phylogeny. A spread of isolation dates for strains of *T. brucei*, *T. congolense*, *T. equiperdum* and *T. vivax* are shown*.* Complete gene coding regions used for time resolved phylogeny are indicated in red. Multiple complete coding regions were obtained for *T. vivax* but clocks were not calculated based on the limited range of isolation dates.

**Additional file 9: Data S2. **Distribution of clock rates sampled from BEAST2 for trypanosome species and subgroups. Nine hundred evenly sampled clock rates from timed phylogeny runs are shown for *T. brucei* (Tb) and the pan-African subgroup, as well for *T. congolense* (Tc) and the savannah subgroup (Tcs). Box and whisker plots show the 10th, 25th, 75th and 90th percentiles with the midline representing the median.

**Additional file 10: Figure S6. **Inferred polyphyly of *T. equiperdum*.T. equiperdum isolates in red font. A maximum likelihood tree inferred from the shared common sequence from the reference sequences of STIB818, STIB841 and STIB842, which have incomplete coding region sequences, and BoTat, Dodola 943 and TeAp ND1, which all have complete maxicircle coding regions. Node values represent bootstrap support.

**Additional file 11.** Additional methods.

**Additional file 12.** Assembled maxicircle FASTA formatted sequence data.

## Data Availability

Public data used to assemble sequences in this study, and the assembled sequences are freely available, and listed by run experiment, Bioproject, and Genbank accession in Additional file 2: Table S1. Assembled sequences used for our analyses are collected together in Additional file 12.

## Ribosomal genes

12S: 12S rRNA
9S: 9S rRNA
RPS12: Ribosomal protein 12

## Respiratory complex genes

ND(1–9): NADH dehydrogenase subunits (1–9)
CYB: Apo-cytochrome *b*
CO(I–III): Cytochrome oxidase subunits (I–III)
A6: ATPase subunit 6

## Genes of unknown function

M(2. 5): Maxicircle Unidentified Reading Frame (MURF, 2, 5)
CR(3, 4): C-rich Reading frame (3, 4
