# Supplementary material for: Mitochondrial DNAs provide insight into trypanosome phylogeny and molecular evolution
Source: BMC Evol Biol. 2020 Dec 9;20:161. doi: 10.1186/s12862-020-01701-9 (PMC7724854; doi:10.1186/s12862-020-01701-9)
Supplement: Supplementary file 2 — Additional file 2: Table S1. Strain information and source data. [file 12862_2020_1701_MOESM2_ESM.docx]

| **Genus** | **Species** | **Subspecies / subgroup** | **Strain** | **Country of isolation** | **Host of isolation** | **Year of isolation** | **Sequence length** | **Data source** | **Bioproject** | **Genbank** |
| --- | --- | --- | --- | --- | --- | --- | --- | --- | --- | --- |
| Crithidia | fasciculata | - | strain Cf-Cl | - | - | - | 29716 |  |  | CM003437.1 |
| Leishmania | tarentolae | - | - | - | - | - | 20991 |  |  | M10126.1 |
| Trypanosoma | brucei | brucei | TREU 927 | Kenya | Tsetse | 1970 | 19354 | ERR027118 | PRJEB2139 |  |
| Trypanosoma | brucei | brucei | Lister 427 var 3 | Kenya | Sheep | 1960 | 18681 | Illumina |  |  |
| Trypanosoma | brucei | brucei | 503 s | Kenya | Bovine | 2017 | 18770 | Illumina |  |  |
| Trypanosoma | brucei | brucei | B8 18 clone B | Nigeria | Pig | 1962 | 18560 | Illumina |  |  |
| Trypanosoma | brucei | brucei | Cow248 | Uganda | Bovine | 2005 | 19672 | Illumina |  |  |
| Trypanosoma | brucei | brucei | Lister 427 | Kenya | Sheep | 1960 | 23016 |  |  | M94286.1 |
| Trypanosoma | brucei | brucei | H879 | Uganda | Bovine | 2009 | 17928 | Illumina |  |  |
| Trypanosoma | brucei | brucei | H883 | Uganda | Canine | 2005 | 17860 | Illumina |  |  |
| Trypanosoma | brucei | brucei | H884 | Uganda | Bovine | 2003 | 17804 | Illumina |  |  |
| Trypanosoma | brucei | brucei | J10 | Zambia | Hyena | 1973 | 18210 | Illumina |  |  |
| Trypanosoma | brucei | brucei | KP33 clone 16 | Côte d'Ivoire | Tsetse | 1989 | 17824 | Illumina |  |  |
| Trypanosoma | brucei | brucei | LF1 | Kenya | Tsetse | 1980 | 18462 | Illumina |  |  |
| Trypanosoma | brucei | brucei | LVBG3N | Kenya | Bovine | 1980 | 17861 | Illumina |  |  |
| Trypanosoma | brucei | brucei | PTAG 129 | Côte d'Ivoire | Pig | 1985 | 17304 | Illumina |  |  |
| Trypanosoma | brucei | brucei | STIB 213 | Tanzania | Hyena | 1971 | 16654 | Illumina |  |  |
| Trypanosoma | brucei | brucei | STIB 348 | Tanzania | Kongoni | 1971 | 20915 | Illumina |  |  |
| Trypanosoma | brucei | brucei | STIB 348TBABB | Tanzania | Hartebeest | - | 20133 | SRS2526092 | PRJNA407592 |  |
| Trypanosoma | brucei | brucei | STIB 776 | Uganda | Tsetse | 1971 | 18744 | Illumina |  |  |
| Trypanosoma | brucei | brucei | STIB 920 | Tanzania | Hartebeest | - | 20207 | SRS2526095 | PRJNA407592 |  |
| Trypanosoma | brucei | brucei | TSW 187 78E | Côte d'Ivoire | Pig | 1978 | 17349 | Illumina |  |  |
| Trypanosoma | brucei | brucei | TSW 55 | Côte d'Ivoire | Pig | 1982 | 18236 | Illumina |  |  |
| Trypanosoma | brucei | gambiense | 141 BT | DR Congo | *Grammomys* | 2005 | 17298 | SRS817052 | PRJNA272153 |  |
| Trypanosoma | brucei | gambiense | 146 AT | DR Congo | *Mastomys* | 2005 | 18154 | SRS817128 | PRJNA272153 |  |
| Trypanosoma | brucei | gambiense | 1829 ALJO | DR Congo | Human | 1970 | 17468 | Illumina |  |  |
| Trypanosoma | brucei | gambiense | FONTEM S10 | Cameroon | Human | 1988 | 18259 | Illumina |  |  |
| Trypanosoma | brucei | gambiense | TH126 | Côte d'Ivoire | Human | 1978 | 18797 | Illumina |  |  |
| Trypanosoma | brucei | rhodesiense | Angwen | Uganda | Human | 2004 | 18402 | SRS2526100 | PRJNA407592 |  |
| Trypanosoma | brucei | rhodesiense | Apendum | Uganda | Human | 2001 | 17590 | SRS2526103 | PRJNA407592 |  |
| Trypanosoma | brucei | rhodesiense | D1 | Kenya | Human | 2009 | 17875 | SRS2526097 | PRJNA407592 |  |
| Trypanosoma | brucei | rhodesiense | D11 | Kenya | Human | 1970 | 20239 | SRS2526096 | PRJNA407592 |  |
| Trypanosoma | brucei | rhodesiense | D2 | Kenya | Human | 1961 | 17904 | SRS2526098 | PRJNA407592 |  |
| Trypanosoma | brucei | rhodesiense | D3 | Kenya | Human | 1969 | 17905 | SRS2526105 | PRJNA407592 |  |
| Trypanosoma | brucei | rhodesiense | D4 | Kenya | Human | 1977 | 17920 | SRS2526104 | PRJNA407592 |  |
| Trypanosoma | brucei | rhodesiense | D5 | Kenya | Human | 1989 | 17862 | SRS2526087 | PRJNA407592 |  |
| Trypanosoma | brucei | rhodesiense | D7 | Kenya | Human | 2000 | 17905 | SRS2526085 | PRJNA407592 |  |
| Trypanosoma | brucei | rhodesiense | Dog157 | Uganda | Dog | 2005 | 17951 | SRS2526088 | PRJNA407592 |  |
| Trypanosoma | brucei | rhodesiense | EATRO 240 | Uganda | Human | 1961 | 20302 | Illumina |  |  |
| Trypanosoma | brucei | rhodesiense | H865 | Uganda | Human | 1990 | 17591 | Illumina |  |  |
| Trypanosoma | brucei | rhodesiense | H866 | Uganda | Human | 1990 | 17861 | Illumina |  |  |
| Trypanosoma | brucei | rhodesiense | KeKo | Kenya | Human | 2012 | 18130 | SRS2526086 | PRJNA407592 |  |
| Trypanosoma | brucei | rhodesiense | LVH 56 | Kenya | Human | 1978 | 17846 | Illumina |  |  |
| Trypanosoma | brucei | rhodesiense | LWO07A | Uganda | Human | 2008 | 17919 | SRS2526082 | PRJNA407592 |  |
| Trypanosoma | brucei | rhodesiense | LWO150A | Uganda | Human | 2010 | 18402 | SRS2526084 | PRJNA407592 |  |
| Trypanosoma | brucei | rhodesiense | LWO24A | Uganda | Human | 2008 | 17860 | SRS2526083 | PRJNA407592 |  |
| Trypanosoma | brucei | rhodesiense | LWO30A | Uganda | Human | 2008 | 17923 | SRS2526090 | PRJNA407592 |  |
| Trypanosoma | brucei | rhodesiense | O58 | Zambia | Human | 1974 | 19805 | Illumina |  |  |
| Trypanosoma | brucei | rhodesiense | Okware | Uganda | Human | 1992 | 17862 | SRS2526089 | PRJNA407592 |  |
| Trypanosoma | brucei | rhodesiense | STIB 324 | Tanzania | Hyena | 1971 | 18328 | Illumina |  |  |
| Trypanosoma | brucei | rhodesiense | STIB 704BA | Tanzania | Human | 1982 | 20335 | Illumina |  |  |
| Trypanosoma | brucei | rhodesiense | STIB 704C | Tanzania | Human | - | 19751 | SRS2526093 | PRJNA407592 |  |
| Trypanosoma | brucei | rhodesiense | STIB 809 | Ethiopia | Human | 1967 | 17903 | Illumina |  |  |
| Trypanosoma | brucei | rhodesiense | STIB 900 | Tanzania | Human | - | 19214 | SRS2526094 | PRJNA407592 |  |
| Trypanosoma | brucei | rhodesiense | YTat 1.1 | Uganda | Human | - | 17906 | SRS2526091 | PRJNA407592 |  |
| Trypanosoma | congolense | BANANCL2 like | BANANCL2 | Burkina Faso | Bovine | 1983 | 14505 | ERS1629274 | PRJEB15251 |  |
| Trypanosoma | congolense | BANANCL2 like | IL3578 | Burkina Faso | Bovine | 1983 | 25468 | ERS1765478 | PRJEB21004 |  |
| Trypanosoma | congolense | forest | DIND | Burkina Faso | Bovine | 1986 | 15307 |  |  |  |
| Trypanosoma | congolense | forest | IL3900 | Burkina Faso | Dog | 1980 | 17531 | ERS1765496 | PRJEB21004 |  |
| Trypanosoma | congolense | kilifi | WG84 | Kenya | Goat | 1981 | 26897 | PacBio |  |  |
| Trypanosoma | congolense | kilifi | ERA D1 | Tanzania | Tsetse | 2006 | 17242 | Illumina |  |  |
| Trypanosoma | congolense | savannah | GAM2 | Gambia | Bovine | 1977 | 20702 | PacBio |  |  |
| Trypanosoma | congolense | savannah | IL1180 | Tanzania | Lion | 1961 | 22008 | ERS1765498 | PRJEB21004 |  |
| Trypanosoma | congolense | savannah | IL2068 | Tanzania | - | 1971 | 21919 | ERS1765480 | PRJEB21004 |  |
| Trypanosoma | congolense | savannah | IL2326 | Uganda | Bovine | 1962 | 17249 | ERS1765483 | PRJEB21004 |  |
| Trypanosoma | congolense | savannah | IL2992 | Kenya | Bovine | 1966 | 25433 | ERS1765445 | PRJEB21004 |  |
| Trypanosoma | congolense | savannah | IL3000 | Kenya | Bovine | 1966 | 27562 | ERS2596592 | PRJEB27608 |  |
| Trypanosoma | congolense | savannah | IL3019 | Kenya | Bovine | 1966 | 25714 | ERS1765459 | PRJEB21004 |  |
| Trypanosoma | congolense | savannah | IL3021 | Kenya | Bovine | 1966 | 26833 | ERS1765444 | PRJEB21004 |  |
| Trypanosoma | congolense | savannah | IL3022 | Kenya | Bovine | 1966 | 26675 | ERS1765456 | PRJEB21004 |  |
| Trypanosoma | congolense | savannah | IL3035 | Kenya | Bovine | 1985 | 26250 | ERS1765448 | PRJEB21004 |  |
| Trypanosoma | congolense | savannah | IL311 | Gambia | Bovine | 1979 | 23812 | ERS1765488 | PRJEB21004 |  |
| Trypanosoma | congolense | savannah | IL3180 | Kenya | Bovine | 1966 | 25382 | ERS1765447 | PRJEB21004 |  |
| Trypanosoma | congolense | savannah | IL3304 | Nigeria | Bovine | 1967 | 22139 | ERS1765500 | PRJEB21004 |  |
| Trypanosoma | congolense | savannah | IL3349 | Kenya | Bovine | 1966 | 21754 | ERS1765485 | PRJEB21004 |  |
| Trypanosoma | congolense | savannah | IL3674 | Gambia | Bovine | 1979 | 25780 | ERS1765492 | PRJEB21004 |  |
| Trypanosoma | congolense | savannah | IL3675 | Gambia | Bovine | 1979 | 24175 | ERS1765467 | PRJEB21004 |  |
| Trypanosoma | congolense | savannah | IL3686 | Kenya | Lion | 1982 | 18515 | ERS1765446 | PRJEB21004 |  |
| Trypanosoma | congolense | savannah | IL3688 | Kenya | Lion | 1982 | 26275 | ERS1765450 | PRJEB21004 |  |
| Trypanosoma | congolense | savannah | IL374 | Kenya | Dog | 1976 | 24183 | ERS1765452 | PRJEB21004 |  |
| Trypanosoma | congolense | savannah | IL3779 | Kenya | Tsetse | 1991 | 21991 | ERS1765491 | PRJEB21004 |  |
| Trypanosoma | congolense | savannah | IL3897 | Burkina Faso | Bovine | 1982 | 26823 | ERS1765463 | PRJEB21004 |  |
| Trypanosoma | congolense | savannah | IL3949 | Kenya | Bovine | 1972 | 21975 | ERS1765465 | PRJEB21004 |  |
| Trypanosoma | congolense | savannah | IL396g | Kenya | Dog | 1976 | 22936 | ERS1765458 | PRJEB21004 |  |
| Trypanosoma | congolense | savannah | IL3978 | - | Bovine | 1992 | 20817 | ERS1765472 | PRJEB21004 |  |
| Trypanosoma | congolense | savannah | IL409 | Kenya | Dog | 1976 | 21606 | ERS1765460 | PRJEB21004 |  |
| Trypanosoma | congolense | savannah | IL438 | Kenya | Dog | 1976 | 20128 | ERS1765489 | PRJEB21004 |  |
| Trypanosoma | congolense | savannah | IL588 | Uganda | Bovine | 1962 | 20991 | ERS1765476 | PRJEB21004 |  |
| Trypanosoma | congolense | savannah | ILC55 | Kenya | Dog | 1976 | 21509 | ERS1765484 | PRJEB21004 |  |
| Trypanosoma | congolense | savannah | KARAN | Burkina Faso | Bovine | 1986 | 23440 | ERS1629286 | PRJEB15251 |  |
| Trypanosoma | congolense | savannah | TRT12 | Zambia | Bovine | 1996 | 22784 | ERS1629307 | PRJEB15251 |  |
| Trypanosoma | congolense | savannah | WG81 | Kenya | Goat | 1981 | 27561 | Illumina |  |  |
| Trypanosoma | congolense | savannah | YATU-F187 | Ethiopia | Bovine | 2010 | 17878 | ERS1629323 | PRJEB27608 |  |
| Trypanosoma | conorhini |  | 025E | Brazil | Rat | 1947 | 17176 |  |  | MKKU01000412.1 |
| Trypanosoma | copemani |  | G1 | Australia | Kangaroo | 2018 | 16450 |  |  | MG948557.1 |
| Trypanosoma | cruzi |  | CL Brener | Brazil | *Triatoma infestans* | 1963 | 20630 |  |  | DQ343645.1 |
| Trypanosoma | cruzi |  | Esmeraldo | Brazil | Human | 1971 | 22295 |  |  | DQ343646.1 |
| Trypanosoma | cruzi |  | Silvio X10 | Brazil | Human | 1983 | 15184 |  |  | FJ203996.1 |
| Trypanosoma | equiperdum |  | BoTat 1 | Morocco | Horse | 1924 | 18213 | SRS2013154 | PRJNA377640 |  |
| Trypanosoma | equiperdum |  | Dodola 943 | Ethiopia | Horse | 2010 | 19336 | SRS2013151 | PRJNA377640 |  |
| Trypanosoma | equiperdum |  | STIB 818 | China | Horse | 1979 | 13541 |  |  | EU185799.1 |
| Trypanosoma | equiperdum |  | STIB 841 |  | Horse |  | 14365 |  |  | HM051262.1 |
| Trypanosoma | equiperdum |  | STIB 842 | China | Horse |  | 14231 |  |  | EU185800.1 |
| Trypanosoma | equiperdum |  | TeAp ND1 | Venezuela | Horse | 2008 | 18649 | SRS2013150 | PRJNA377640 |  |
| Trypanosoma | godfreyi |  | ERA F1 | Tanzania | Tsetse | 2006 | 17422 | Illumina |  |  |
| Trypanosoma | godfreyi |  | KEN7 | Gambia | Tsetse | 1988 | 23357 | PacBio |  |  |
| Trypanosoma | grayi |  | ANR4 | Gambia | Tsetse | 1991 | 15558 | SRS644817 | PRJNA244495 |  |
| Trypanosoma | lewisi |  |  |  |  |  | 23754 |  |  | KR072974.1 |
| Trypanosoma | simiae |  | ERA C2 | Tanzania | Tsetse | 2006 | 22119 | Pacbio |  |  |
| Trypanosoma | simiae | tsavo | KETRI 3436 | Kenya | Pig | 1971 | 17134 | Illumina |  |  |
| Trypanosoma | theileri |  | Edinburgh TM35 |  |  |  | 36120 | NBCO01000000 | PRJNA273795 |  |
| Trypanosoma | vivax |  | Liem 176 | Venezuela | Bovine |  | 15330 | KM386509.1 |  |  |
| Trypanosoma | vivax |  | MT1 | Venezuela | Bovine |  | 19082 | KM386508.1 |  |  |
| Trypanosoma | vivax |  | Tv1392 | Nigeria | Bovine | 1981 | 17521 | SRS3670567 | PRJNA486085 |  |
| Trypanosoma | vivax |  | Tv2005 | Uganda | Bovine | 1969 | 16328 | SRS3670558 | PRJNA486085 |  |
| Trypanosoma | vivax |  | Tv2323 | Uganda | Bovine | 1969 | 16598 | SRS3670561 | PRJNA486085 |  |
| Trypanosoma | vivax |  | Tv2714 | Uganda | Bovine | 1969 | 16412 | SRS3670557 | PRJNA486085 |  |
| Trypanosoma | vivax |  | Tv306 | Nigeria | Bovine | 1973 | 16186 | SRS3670566 | PRJNA486085 |  |
| Trypanosoma | vivax |  | Tv3171 | Gambia | Bovine | 1973 | 16776 | SRS3670565 | PRJNA486085 |  |
| Trypanosoma | vivax |  | Tv319 | Nigeria | Bovine | 1973 | 17632 | SRS3670562 | PRJNA486085 |  |
| Trypanosoma | vivax |  | Tv340 | Nigeria | Bovine | 1962 | 15825 | SRS3670560 | PRJNA486085 |  |
| Trypanosoma | vivax |  | Tv3638 | Côte d'Ivoire | Bovine | 1990 | 16797 | SRS3670570 | PRJNA486085 |  |
| Trypanosoma | vivax |  | Tv3651 | Côte d'Ivoire | Bovine | 1990 | 17087 | SRS3670575 | PRJNA486085 |  |
| Trypanosoma | vivax |  | Tv3658 | Côte d'Ivoire | Bovine | 1990 | 16307 | SRS3670576 | PRJNA486085 |  |
| Trypanosoma | vivax |  | Tv462 | Nigeria | Bovine | 1973 | 16151 | SRS3670569 | PRJNA486085 |  |
| Trypanosoma | vivax |  | Tv493 | Nigeria | Bovine | 1973 | 17978 | SRS3670574 | PRJNA486085 |  |
| Trypanosoma | vivax |  | Tv596 | Nigeria | Bovine | 1973 | 18324 | SRS3670573 | PRJNA486085 |  |
| Trypanosoma | vivax |  | Tv684 | Nigeria | Bovine | 1973 | 16419 | SRS3670572 | PRJNA486085 |  |
| Trypanosoma | vivax |  | Y486 | Nigeria | Bovine | 1976 | 19833 | ftp://ftp.sanger.ac.uk/pub/project/pathogens/Trypanosoma/vivax | |  |

**Table S1. Strain information and source data.**

Reference and assembled trypanosome mitochondrial DNA sequences are presented here along with strain isolation information. For sequences assembled from resources in the SRA archive, run experiment and Bioproject are listed. In all 9 different projects contributed to our assembled maxicircle collection.
