# Supplementary material for: Mitochondrial DNAs provide insight into trypanosome phylogeny and molecular evolution
Source: BMC Evol Biol. 2020 Dec 9;20:161. doi: 10.1186/s12862-020-01701-9 (PMC7724854; doi:10.1186/s12862-020-01701-9)
Supplement: Supplementary file 3 — Additional file 3: Figure S2. Assembled variable regions of salivarian maxicircle mitochondrial DNAs. [file 12862_2020_1701_MOESM3_ESM.pdf]

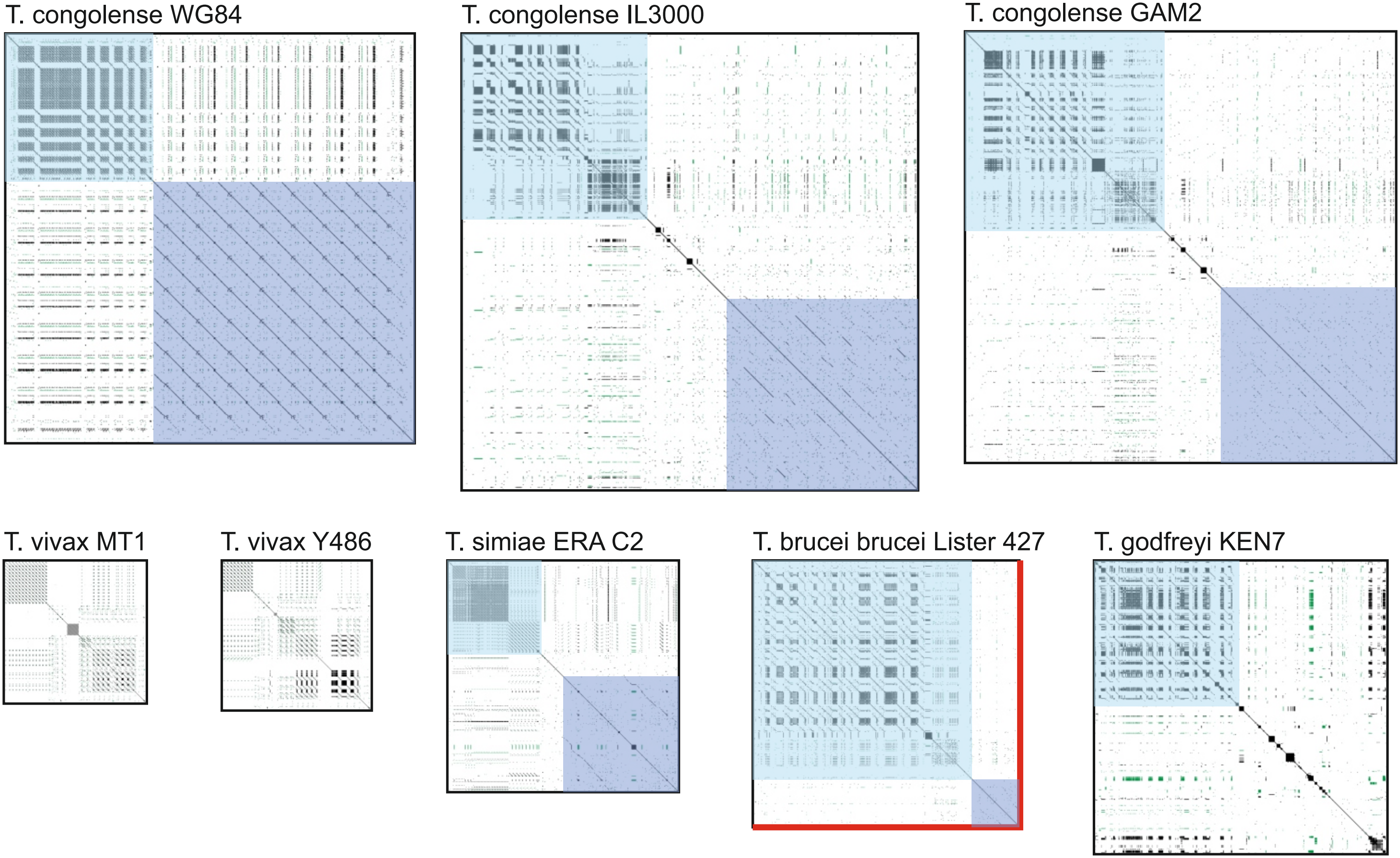
T. congolense WG84
T. congolense IL3000
T. congolense GAM2
T. vivax MT1
T. vivax Y486
T. simiae ERA C2
T. brucei brucei Lister 427
T. godfreyi KEN7
