# Supplementary figures and images for: Mitochondrial DNAs provide insight into trypanosome phylogeny and molecular evolution
Source: BMC Evol Biol. 2020 Dec 9;20:161. doi: 10.1186/s12862-020-01701-9 (PMC7724854; doi:10.1186/s12862-020-01701-9)

m54053\_181105\_161246

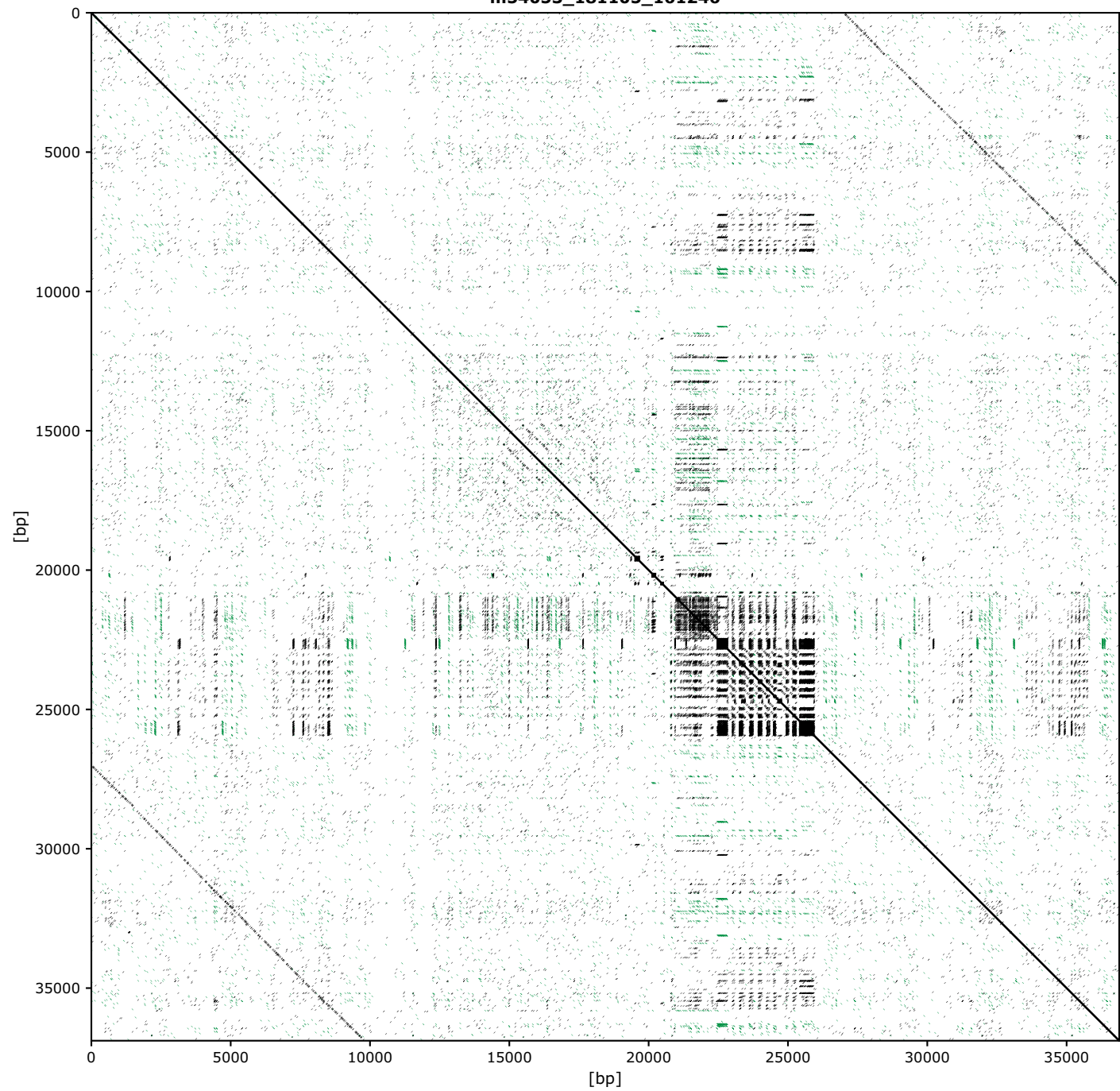

Supplement: Supplementary file 4 — Additional file 4: Figure S1. An example of a PacBio read spanning the entire sequence of the trypanosome mitochondrial DNA (maxicircle). A single read from the T. congolense GAM2 readpool is shown dot-plotted against itself. The highly repetitive short period portion of the variable region is visualised as a densely self-similar region between 20-26 kbp, whilst the longer period portion of the variable region begins at 15 kbp. The remainder of the sequence shown belongs to the gene coding region. The complete length of the maxicircle is seen from 0-26.3 kbp, and thereafter begins to repeat. The assembled sequence is shown in Additional file 3: Figure S2. [file 12862_2020_1701_MOESM4_ESM.pdf]

ND7

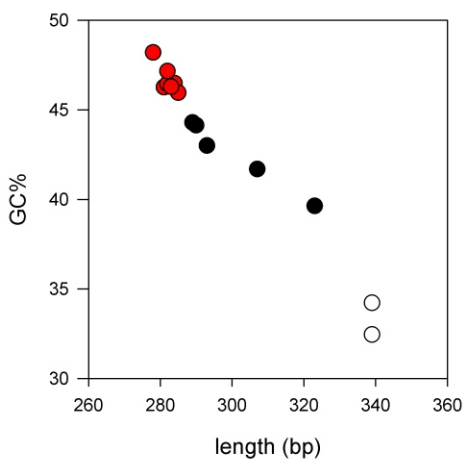

ND8

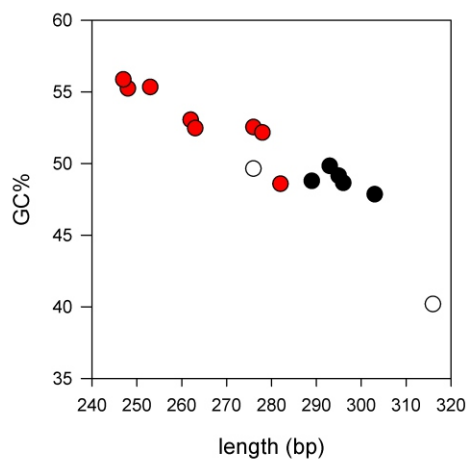

ND9

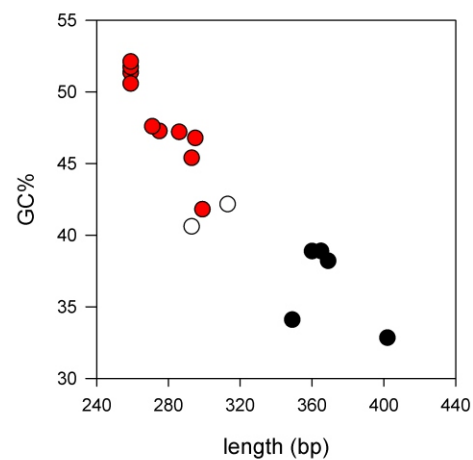

CO3

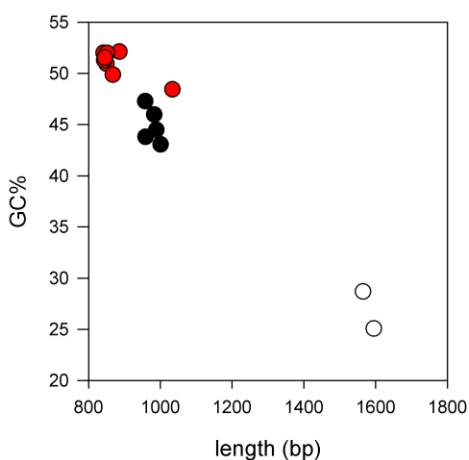

A6

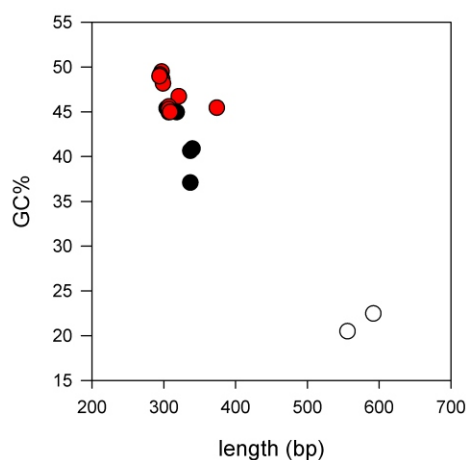

CR3

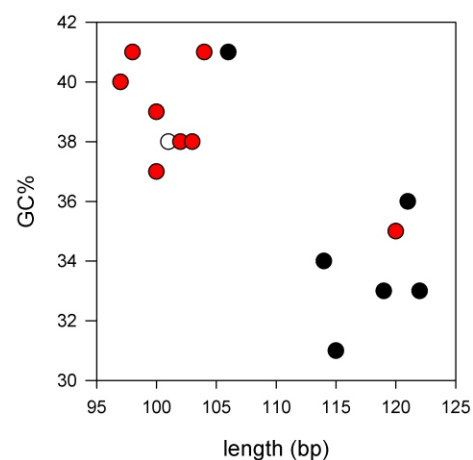

ND7

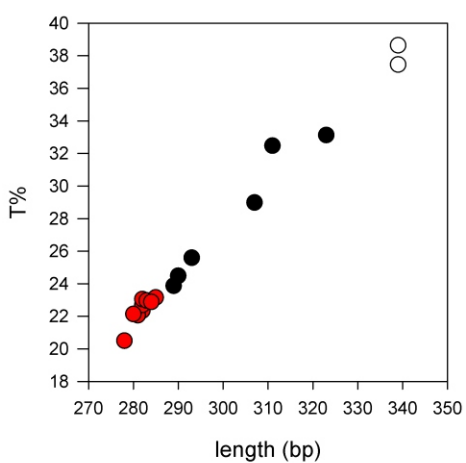

ND8

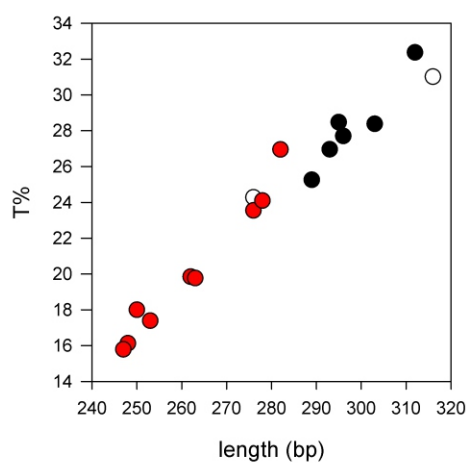

ND9

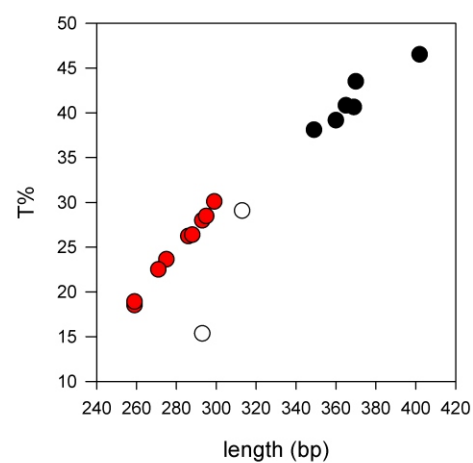

CO3

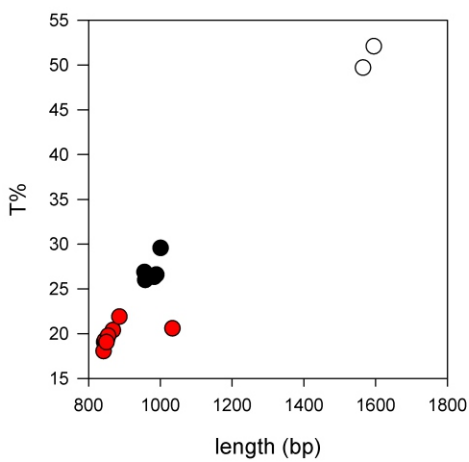

A6

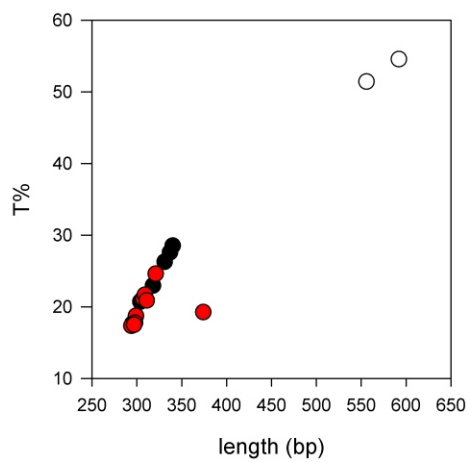

CR3

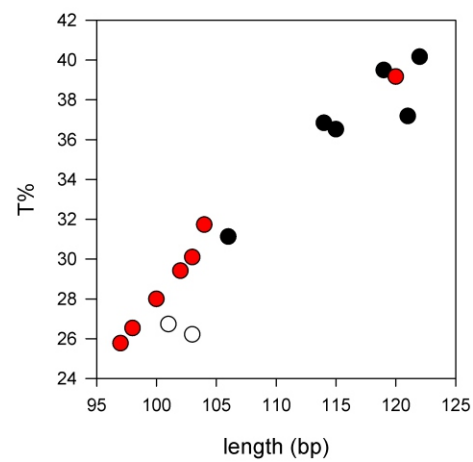

Supplement: Supplementary file 6 — Additional file 6: Figure S4. Correlation between sequence length, GC% and T% for six pre-edited maxicircle genes. Some pre-edited maxicircle genes exhibit transcript length variation with strong correlation between length and T% as well as an inverse correlation for GC%. The weak negative correlation for A% indicates that this is a strand specific phenomenon consistent with RNA editing, where uridines are inserted back into the transcript. Key: Crithidia, Leishmania (open circle), salivarian (red filled circle) and non-salivarian trypanosomes (black filled circle). [file 12862_2020_1701_MOESM6_ESM.pdf]

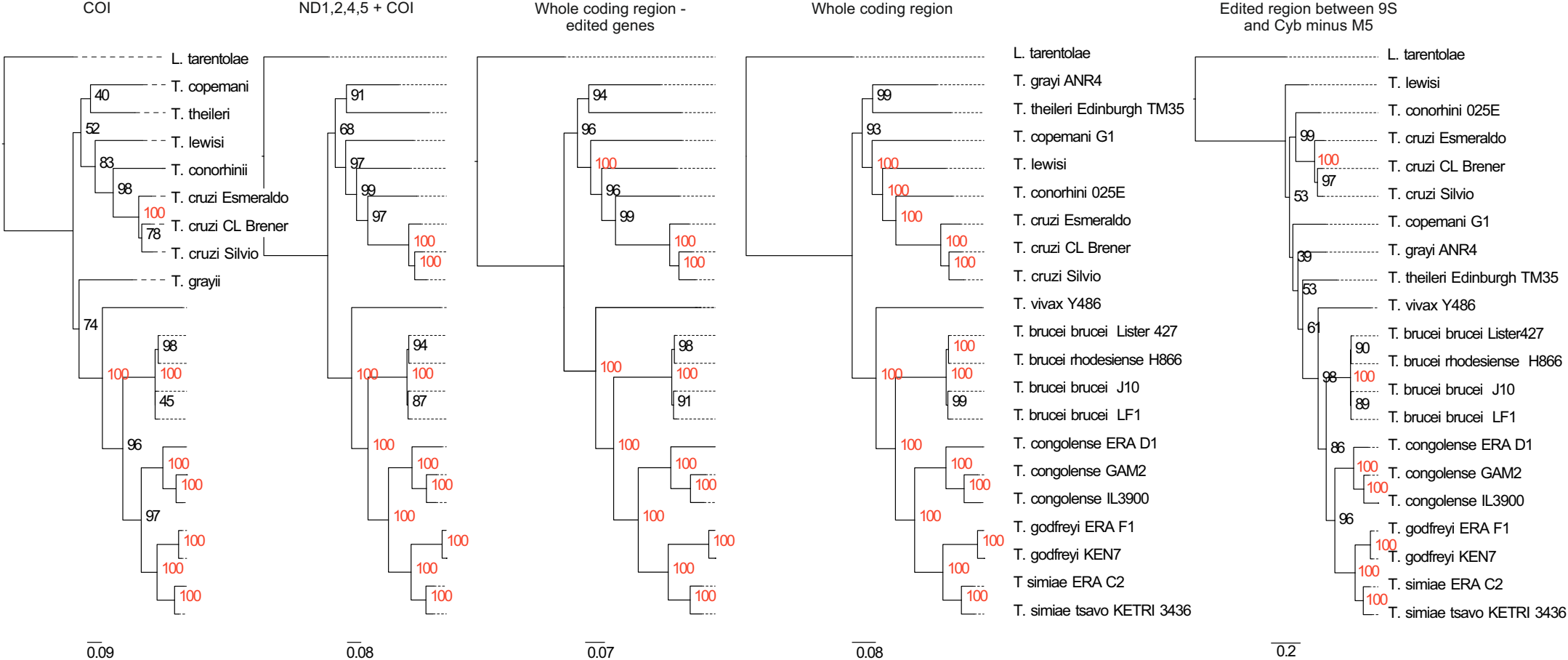

Supplement: Supplementary file 7 — Additional file 7: Figure S5. A comparison of maximum likelihood trees inferred from different regions of the trypanosome maxicircle mitochondrial DNA. In general using more sequence contributes to higher bootstrap support for the inferred maximum likelihood topology. If individual genes are used, confidence for the deepest branches is reduced, and topological variances are observed. Collections of non-edited genes have a consistent topology but fail to resolve well within species. Use of the entire gene coding region (WCR), with or without pre-edited genes, provides better supported trees. If pre-edited genes alone are used, structure within species is well supported, but multispecies relationships are poorly resolved. [file 12862_2020_1701_MOESM7_ESM.pdf]

# Comparison of sampled clock rates

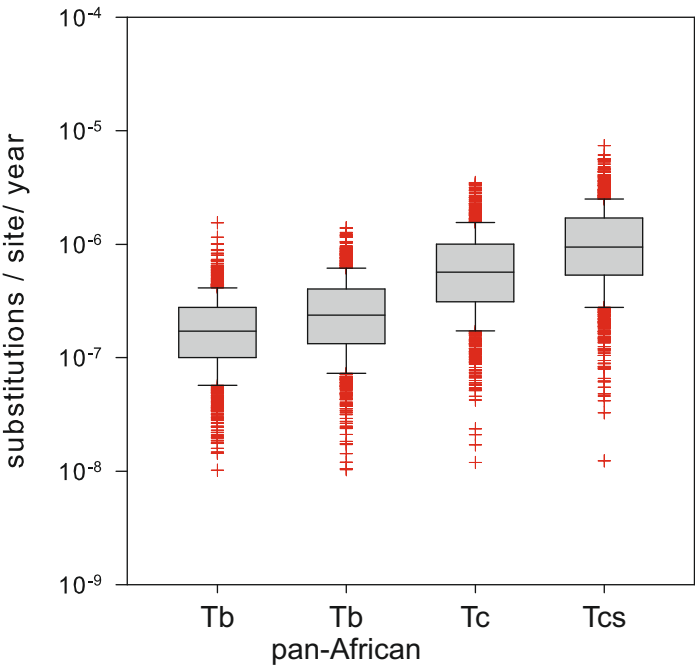

Supplement: Supplementary file 8 — Additional file 8: Data S1. Distribution of isolation dates used for inferring time-resolved phylogeny. A spread of isolation dates for strains of T. brucei, T. congolense, T. equiperdum and T. vivax are shown. Complete gene coding regions used for time resolved phylogeny are indicated in red. Multiple complete coding regions were obtained for T. vivax but clocks were not calculated based on the limited range of isolation dates. [file 12862_2020_1701_MOESM8_ESM.pdf]

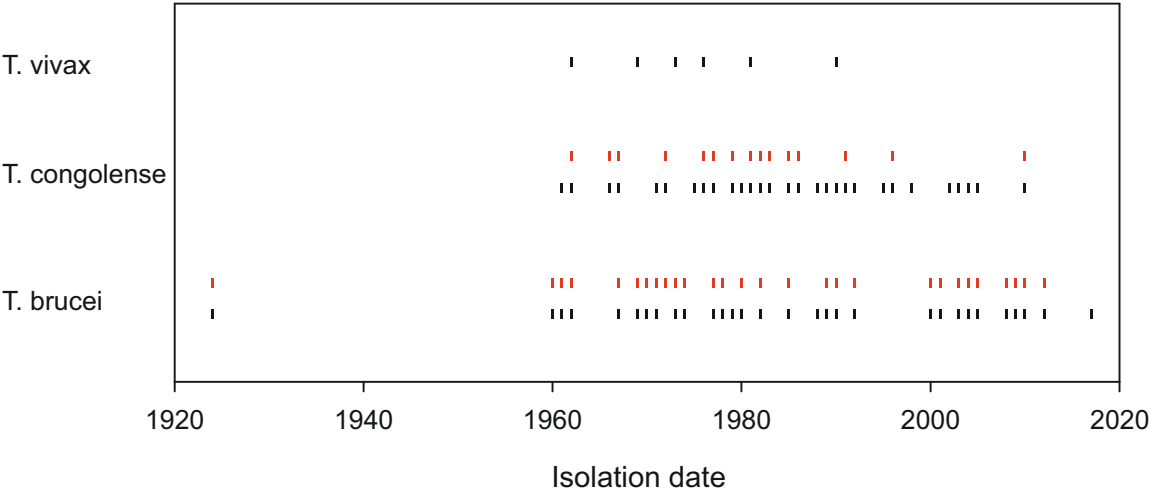

Supplement: Supplementary file 9 — Additional file 9: Data S2. Distribution of clock rates sampled from BEAST2 for trypanosome species and subgroups. Nine hundred evenly sampled clock rates from timed phylogeny runs are shown for T. brucei (Tb) and the pan-African subgroup, as well for T. congolense (Tc) and the savannah subgroup (Tcs). Box and whisker plots show the 10th, 25th, 75th and 90th percentiles with the midline representing the median. [file 12862_2020_1701_MOESM9_ESM.pdf]

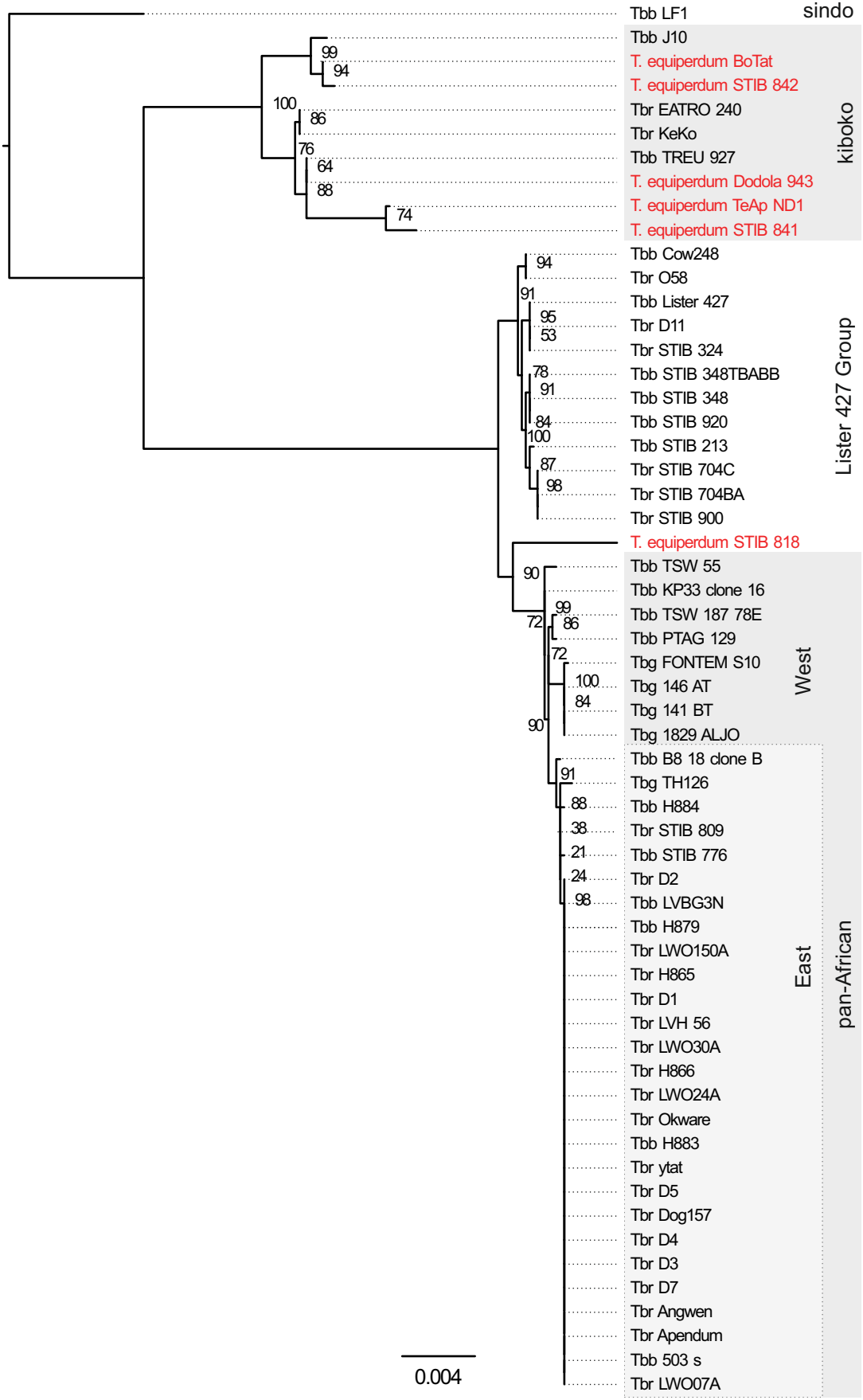

Supplement: Supplementary file 10 — Additional file 10: Figure S6. Inferred polyphyly of T. equiperdum.T. equiperdum isolates in red font. A maximum likelihood tree inferred from the shared common sequence from the reference sequences of STIB818, STIB841 and STIB842, which have incomplete coding region sequences, and BoTat, Dodola 943 and TeAp ND1, which all have complete maxicircle coding regions. Node values represent bootstrap support. [file 12862_2020_1701_MOESM10_ESM.pdf]
