# Supplementary material for: Mitochondrial DNAs provide insight into trypanosome phylogeny and molecular evolution
Source: BMC Evol Biol. 2020 Dec 9;20:161. doi: 10.1186/s12862-020-01701-9 (PMC7724854; doi:10.1186/s12862-020-01701-9)
Supplement: Supplementary file 5 — Additional file 5: Figure S3. Trypanosoma vivax, an alignment of the intergenic region between 9S and ND8 containing a putative microsatellite. Bases are shown as coloured bands with the top line tick showing 20 bp increments. The sequence [ATATA] is tandemly repeated between 18 and 51 times in the selected isolates. [file 12862_2020_1701_MOESM5_ESM.pdf]

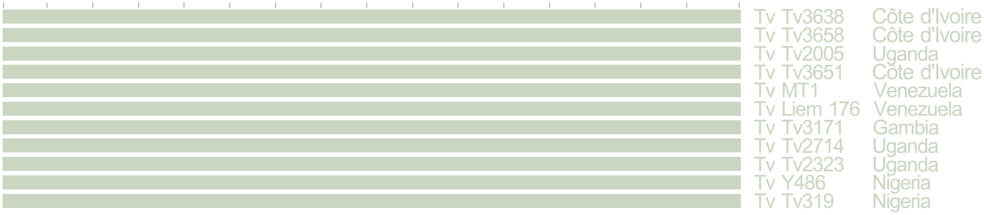
Tv Tv3638 Côte d'Ivoire
Tv Tv3658 Côte d'Ivoire
Tv Tv2005 Uganda
Tv Tv3651 Côte d'Ivoire
Tv MT1 Venezuela
Tv Liem 176 Venezuela
Tv Tv3171 Gambia
Tv Tv2714 Uganda
Tv Tv2323 Uganda
Tv Y486 Nigeria
Tv Tv319 Nigeria
