# Supplementary material for: Mitochondrial DNAs provide insight into trypanosome phylogeny and molecular evolution
Source: BMC Evol Biol. 2020 Dec 9;20:161. doi: 10.1186/s12862-020-01701-9 (PMC7724854; doi:10.1186/s12862-020-01701-9)
Supplement: Supplementary file 11 — Additional file 11. Additional methods. [file 12862_2020_1701_MOESM11_ESM.docx]

**Reference Illumina assembly**

Where reference maxicircles were available, assembly of maxicircles from SRA data proceeded as follows:

1. A BLAST database was constructed of a duplicated complete maxicircle to help get even read coverage

<-------maxicircle------><-------maxicircle------>

<--read-->

1. Magic-BLAST was then used to retrieve mapping reads, using the -no_unaligned -paired -limit_lookup false -validate_seqs false arguments
2. Read headers were then extracted from the result sam file, and reads (fastq) retrieved using GNU parallel
3. SPAdes was then used to assemble maxicircle sequences using the default settings for paired read data.
4. Assembled contigs were then BLASTed against the reference genome, only large complete contigs were considered in this study, but smaller contigs could be ordered and scaffolded using MeDuSa.

**NOVOplasty**

Where no close reference maxicircle was available, gene coding regions could be obtained through extension of a conserved gene (typically COI). NOVOplasty was used for this task with these user selected settings in the config file. Insert size was varied according to illumina run.

Type = mito

Genome Range = 20000-50000

K-mer = 25

Platform = illumina

Single/Paired = PE

Insert size auto = yes

Insert Range = 1.9

Insert Range strict = 1.3

Use Quality Scores = no

Extended seed regions were then used as references for SPAdes assemblies of related isolates as described above.

**Pacbio assembly**

Assembly is similar as detailed above and consists of these principle steps:

1. A BLAST database was constructed of a related maxicircle sequence
2. Pacbio reads were then BLASTed against this database
3. Read sequences with an alignment length of greater than 1000bp were kept
4. Reads identified from this first round were used to fish out aligning reads, the number of iterations required depended on the average pacbio read length, in our data we were fortunate to have maxicircle spanning guide reads so this iteration was not required more than once.
5. Reads were then corrected using canu using the arguments

canu -pacbio-raw -genomeSize=20k -correct

1. Some guide reads encode complete or partial repeats of the maxicircle sequence, and we found these to be disruptive to assembly. Canu corrected reads were sliced to a maximum length of 12kbp before assembly with Flye (using genome size 25kbp and pacbio-corrected reads as input arguments). We found that canu could not assemble maxicircle sequences due to internal read trimming processes.
2. Maxicircles were then identified by BLAST
3. Raw reads were then aligned to the assembled maxicircle contigs using BWA

In this study pacbio data was collected from species with no close related sequence, and so direct read mapping was not considered.

**Sanger assembly**

1. Similar to pacbio assembly
2. A BLAST database of a related maxicircle was created
3. Aligning Sanger sequencing reads were filtered by BLAST with an alignment length >300bp
4. Filtered reads were than assembled with CAP3 on default settings

**Reducible content**

#! /bin/bash

# read input fasta $1

# Assumes 2 line FASTA

# Assumes ORFs with no gaps, starting in frame with the beginning of the line

# Remove path

intermediate=${1##*/};

# Get base name

name=${intermediate%%.*};

# Make folder from base name

mkdir ./$name

# Write results headers to output file

echo -e "strain\tcodons\treducible_condon_count\tunreducible_condon_count" >> ./$name/"reducible_CG_content.txt"

# For each ORF

while read line; do

# If header line

if [[ ${line:0:1} == '>' ]]

then

# Reset variables

((reducible_condon_count=0))

((unreducible_condon_count=0))

((codons=0))

# Get the associated ORF sequence

sequence=$(grep -A 1 $line $1 | tail -1)

# Work through the sequence length in codons/triplets

for (( i=0; i<${#sequence}; i=i+3 )); do

# Get codon

char=${sequence:$i:3};

#echo $char

# These are the GC reducible codons for this system, yours may vary

if [[ $char == 'TAG' || $char == 'GCC' || $char == 'GCG' || $char == 'TGC' || $char == 'GAC' || $char == 'GAG' || $char == 'TTC' || $char == 'GGC' || $char == 'GGG' || $char == 'CAC' || $char == 'ATC' || $char == 'AAG' || $char == 'TTG' || $char == 'CTC' || $char == 'CTG' || $char == 'AAC' || $char == 'CCC' || $char == 'CCG' || $char == 'CAG' || $char == 'TAC' || $char == 'CGC' || $char == 'CGA' || $char == 'CGG' || $char == 'AGG' || $char == 'TCC' || $char == 'TCG' || $char == 'AGC' || $char == 'ACC' || $char == 'GTC' || $char == 'GTG' || $char == 'TGG' ]]

then

((reducible_condon_count++))

((codons++))

else

((unreducible_condon_count++))

((codons++))

fi

done

# Write out results

echo -e $line "\t" $codons "\t" $reducible_condon_count "\t" $unreducible_condon_count >> ./$name/"reducible_CG_content.txt"

fi

done < $1

**GC-less codon determination**

#! /bin/bash

# read input fasta

intermediate=${1##*/};

name=${intermediate%%.*};

mkdir ./$name

echo -e "strain\tcodons\treducible_condon_count\tunreducible_condon_count" >> ./$name/"reducible_CG_content.txt"

while read line; do

# if header line

if [[ ${line:0:1} == '>' ]]

then

((reducible_condon_count=0))

((unreducible_condon_count=0))

((codons=0))

sequence=$(grep -A 1 $line $1 | tail -1)

for (( i=0; i<${#sequence}; i=i+3 )); do

char=${sequence:$i:3}

echo $char

if [[ $char == 'TTT' || $char == 'ATT' || $char == 'ATA' || $char == 'AAA' || $char == 'TTA' || $char == 'AAT' || $char == 'TAT' ]]

then

((reducible_condon_count++))

((codons++))

else

((unreducible_condon_count++))

((codons++))

fi

done

echo -e $line "\t" $codons "\t" $reducible_condon_count "\t" $unreducible_condon_count >> ./$name/"reducible_CG_content.txt"

fi

done < $1
